# Beneficial modulation of human health in the oral cavity and beyond using bacteriocin-like inhibitory substance-producing streptococcal probiotics

**DOI:** 10.3389/fmicb.2023.1161155

**Published:** 2023-03-28

**Authors:** John R. Tagg, Liam K. Harold, Rohit Jain, John D. F. Hale

**Affiliations:** Blis Technologies Ltd., South Dunedin, New Zealand

**Keywords:** probiotics, BLIS, bacteriocins, oral cavity, *Streptococcus salivarius*, oral health, dental caries, halitosis

## Abstract

The human oral cavity contains a diversity of microbial habitats that have been adopted and adapted to as homeland by an amazingly heterogeneous population of microorganisms collectively referred to as the oral microbiota. These microbes generally co-habit in harmonious homeostasis. However, under conditions of imposed stress, as with changes to the host’s physiology or nutritional status, or as a response to foreign microbial or antimicrobial incursions, some components of the oral “microbiome” (viz. the *in situ* microbiota) may enter a dysbiotic state. This microbiome dysbiosis can manifest in a variety of guises including streptococcal sore throats, dental caries, oral thrush, halitosis and periodontal disease. Most of the strategies currently available for the management or treatment of microbial diseases of the oral cavity focus on the repetitive “broad sweep” and short-term culling of oral microbe populations, hopefully including the perceived principal pathogens. Both physical and chemical techniques are used. However, the application of more focused approaches to the harnessing or elimination of key oral cavity pathogens is now feasible through the use of probiotic strains that are naturally adapted for oral cavity colonization and also are equipped to produce anti-competitor molecules such as the bacteriocins and bacteriocin-like inhibitory substances (viz BLIS). Some of these probiotics are capable of suppressing the proliferation of a variety of recognized microbial pathogens of the human mouth, thereby assisting with the restoration of oral microbiome homeostasis. BLIS K12 and BLIS M18, the progenitors of the BLIS-producing oral probiotics, are members of the human oral cavity commensal species *Streptococcus salivarius.* More recently however, a number of other streptococcal and some non-streptococcal candidate oral probiotics have also been promoted. What is becoming increasingly apparent is that the future for oral probiotic applications will probably extend well beyond the attempted limitation of the direct pathological consequences of oral microbiome dysbiosis to also encompass a plethora of systemic diseases and disorders of the human host. The background to and the evolving prospects for the beneficial modulation of the oral microbiome *via* the application of BLIS-producing *S. salivarius* probiotics comprises the principal focus of the present review.

## The human oral cavity as a microbial habitat

The indigenous microbes of the oral cavity are predominantly and distinctively site-specific in their habitat, their tropisms being determined by characteristic physical, physiological, and biological compatibilities that are the outcome of thousands of years of co-evolution with their human host. Interestingly the *in vogue* descriptor for the oral microbial community has progressively evolved over the years from “oral microflora” to “oral microbiota” to the presently favored “oral microbiome.” Every human harbors a personalized oral cavity microbiome and its role when present in finely balanced equilibrium is critical for health maintenance. Unfortunately, at times, our oral microbiome can become destabilized in the face of therapeutic, dietary or host physiological changes and this dysbiosis can become a source of harm to host tissues, both oral and systemic.

A core human microbiome seems likely to be common to all individuals (Turnbaugh et al., 2007). The human oral microbiome database (Gao et al., 2018) lists approximately 700 bacterial species, only half of which are at present officially named. Included are 43 *Streptococcus* species, 26 of which are named. As the largest bacterial group in the oral cavity the streptococci inevitably are a significant contributor to our oral health (Dewhirst et al., 2010). The core oral microbiome develops as a direct response to a unique combination of host lifestyle and genetic determinants and the essence of its composition is likely to be as specific to ourselves as is our fingerprint (McLean et al., 2022). The foundations of the oral microbiome are established in the perinatal period and comprise an impressive repertoire of microbes that are indigenous to this habitat (Kaan et al., 2021; Kageyama et al., 2022). At birth, the baby may harbor some microbes obtained from the mother during passage through the birth canal, but soon will also acquire from its various close contacts a rich menagerie of attached, planktonic and intracellular microbes. The pioneer colonizing microbes mirror those of the parents-especially the mother (Carlsson et al., 1970; Tagg et al., 1983). Fine-tuning of the microbiome however, will continue throughout life in response to physiological changes and natural aging processes and sometimes it will incur major (potentially detrimental) population shifts (dysbiosis) following host disease, major dietary shifts or antibiotic interventions.

The oral cavity is the body’s principal entry portal for novel microbes and each day a myriad will enter from the environment or from other animal contacts (mostly human) seeking a suitable niche for multiplication of their own kind. Some of these microbes upon entering the oral cavity will attach, some will invade, but most pass through unimpeded–and all (or their products) potentially contribute to the ecology of the oral microbiome. Planktonic bacterial cells may attach either directly or to saliva-coated oral cavity surfaces or indirectly *via* binding to other bacterial cells that have already colonized (Kolenbrander et al., 2002). Dental biofilms are multispecies ecosystems in which various oral species, tethered by salivary proteins and microbial exopolysaccharides, can interact cooperatively or competitively. Biofilms are able to resist some mechanical stress and their inhabitants can often shun antimicrobial treatments. Adhesion *via* coaggregation can be critical for the early retention of newly-introduced microbes on surfaces and may facilitate their subsequent colonization. Communication within the biofilm occurs *via* a number of mechanisms including metabolic synergies, genetic exchanges, inhibitor interactions, and quorum sensing, the later occurring in response to cell density changes and potentially having an influence both on virulence and bacteriocin production (Sedgley et al., 2008; Mignolet et al., 2018, 2019; Hols et al., 2019). Although biofilm-located microbes appear relatively refractory to the action of most chemotherapeutic agents there are indications that some bacteriocins may have efficacy for the treatment of biofilms, especially when these are used in combination with other agents known to function as stressors for the cell envelopes of planktonic cells (Mathur et al., 2018).

Increased knowledge of the principles of microbial ecology has heightened our awareness of the importance of the intimate and interdependent relationships between the human host and its oral microbiome. Subtle changes within the microbiome environment can directly influence gene expression, metabolic activity, inter-microbial competitiveness and ultimately the composition of the microbiome–and this in turn may have significant consequences for the physical health and perhaps even the emotional wellbeing of the host. Probiotic incursions within the resident oral cavity microbiome have historically largely been in the form of the fleeting passage of intestinal probiotics *en route* to the gastrointestinal tract. It has been only relatively recently that purposeful probiotic interventions have been developed using oral cavity sourced lineages of microorganisms such as the BLIS-producing *S. salivarius* probiotics that are already naturally pre-programmed for functional residence within the human oral cavity.

## Some terminology updates

### Microbiota and microbiome

The human microbiota is the collection of microorganisms, predominantly bacteria–but also including minority populations of archaea, fungi, viruses, mycoplasma, and protozoa that live in intimate association with the human body (Li X. et al., 2022). The term refers to the ecological community of commensal, symbiotic, and pathogenic microorganisms that are now recognized to be major determinants of both our health and disease (Lederberg and McCray, 2001). The distinction between the terms microbiome and microbiota has often been misunderstood. However, one contemporary view is that the microbiome encapsulates the microbiota (the community of microorganisms) together with their “theater of activity,” the latter comprising the microbial structural elements, metabolites/signal molecules and the surrounding environmental conditions (Berg et al., 2020). The microbiome provides various traits that humans have not been required to evolve for themselves. Indeed, the expression of the human metagenome (the genome of the entire human microbiome) provides a wide variety of important resources not associated with human cellular activity. Sleator has used the descriptor “human superorganism” to refer to the communal group of cells (only ca. 10% of which are human) that (largely) work in synchrony for the benefit of the collective entity—i.e., a fully-functional human being (Sleator, 2010). Other researchers favor the use of the term “human supraorganism” (Turnbaugh et al., 2007). Hill (2021) has promoted the concept that the human microbiome be considered to be an essential virtual organ, the integrity of which should be protected in the face of therapeutic (e.g., broad-spectrum antibiotic) interventions. This supports the value of adopting more selective (or targeted) microbial culling strategies using relatively narrow spectrum inhibitors such as the bacteriocins and BLIS to suppress pathogen numbers within the microbiome. By minimizing disruption to the existing microbiome composition, the proliferation of disease-initiating outgrowths of potentially pathogenic microbes (such as those present in a “carriage” state) is inhibited.

### Dysbiosis

Dysbiosis can be viewed as a reduction in microbial diversity associated with a loss of beneficial microbes and an increase in pathobionts (viz. organisms that are typically indigenous to a microbiota, but sometimes becoming pathogenic and contributing to disease). Dysbiosis typically ensues either following or in association with disruption to microbial homeostasis due to an imbalance within the microbiome resulting either from underlying compositional or metabolic changes. Some factors triggering dysbiosis within the oral microbiome include antibiotic medications, dietary shifts (e.g., elevated sucrose or alcohol intake), a reduction in saliva flow and a proliferation of microbial pathogens. Many dental diseases and also an increasing number of systemic diseases of the human host (Peng et al., 2022; Xu et al., 2022) are now considered to be a consequence of dysbiotic shifts within the normally stable resident oral microbiome (Radaic and Kapila, 2021). The fostering of populations of health-enhancing species and functions *via* the judicious application of probiotics can help to mitigate against disease expression (Hoare et al., 2017; Rosier et al., 2017).

### Probiotics

Although the formal definition of probiotic (*viz.* “pro-life”) has undergone a series of evolutionary changes over the years, the contemporary view is that probiotics are “live microorganisms that, when administered in adequate amounts, confer a health benefit to the host,” a term adopted by the World Health Organisation (WHO) (Reid, 2005; Teughels et al., 2008). The associated guidelines for use of the term “probiotic” stipulate the need for both a specific microbial strain designation and for at least one study demonstrating a health benefit to be performed in the target host species in order for the strain to be appropriately referred to as a probiotic. Additionally, more specific guidelines for the scientifically acceptable application of the term probiotic to a microbe have also been proposed (Hill et al., 2014). Unfortunately, these guidelines are not always adhered to and all too often candidate strains are prematurely promoted as probiotics prior to the demonstration that their administration actually confers a health benefit to the proposed target species (Reid et al., 2019). Use of the qualifier term “potential” or “putative” should always be adopted until proof of probiotic efficacy is obtained. As the public acceptance and adoption of probiotics continues to escalate, it remains of paramount importance to give stringent regulatory attention to the building of robust safety profiles for all candidate strains (van den Nieuwboer and Claassen, 2019).

It is now evident that the beneficial actions of probiotics can be attributed to a variety of factors including their metabolic characteristics, the molecules presented on the cell surface and their secreted products. Integral cell components such as the DNA and peptidoglycan may also contribute toward probiotic efficacy (Bermudez-Brito et al., 2012). Important attributes for any microbe proposed for use as a probiotic in humans include: (a) relatively targeted anti-pathogen activity (b) absence of cytotoxicity for human tissues (c) being of a species that is consistently prevalent within the human microbiota and (d) exhibiting a capability of integrating and persisting within the human microbiome.

Beneficial outcomes from the use of probiotics are principally derived from their:

(1) Modulation of the host immune system (Raheem et al., 2021).
(2) Molecular interactions with other microbes (commensals or pathogens) (Mignolet et al., 2018, 2019; Hols et al., 2019).
(3) Influences on microbial products (e.g., toxins), host cell products (e.g., bile salts) or food components (e.g., *via* enzyme activity) (Petrova et al., 2022).

### Prebiotics, synbiotics, postbiotics, and oral probiotics

#### Prebiotics

Prebiotics were instigated by Gibson and Roberfroid (1995) as a dietary means of altering the human colonic microbiota toward a more favorable community structure. The International Scientific Association for Probiotics and Prebiotics (ISAPP) has published a consensus view on prebiotics refining the definition to “a substrate that is selectively utilized by host microorganisms, conferring a health benefit” (Gibson et al., 2017). This updated definition now includes provision for non-carbohydrate compounds, and the site of their action is not limited to the gastrointestinal tract, nor is its type limited to food. Researchers are increasingly exploring the potential roles of prebiotics in increasing the beneficial activities of oral probiotics and in modulating the composition and host interactivity of the oral microbiome (Devine and Marsh, 2009).

#### Synbiotics

Synbiotics were initially conceived of as a combination of probiotics and prebiotics and are now defined as “a mixture comprising live microorganisms and substrate(s) selectively utilized by host microorganisms that confers a health benefit on the host” (Swanson et al., 2020). A synergistic synbiotic is a synbiotic for which the substrate is designed to be selectively utilized by the co-administered microorganisms (Swanson et al., 2020). Simply put, the microbial component does not necessarily have to be a stand-alone probiotic and the non-digestible substrate does not necessarily have to be a stand-alone prebiotic, but, if together they provide a health benefit, then the mixture can be called a synbiotic.

#### Postbiotics

In 2019 an ISAPP expert panel proposed a consensus definition of postbiotics as “preparations of inanimate microorganisms and/or their components that confer a health benefit on the host” (Salminen et al., 2021b). Postbiotics contain inactivated microbial cells or cell components, with or without their metabolites, and these must be demonstrated to contribute to observed health benefits in the target host (species and subpopulation). The panel also noted that some confusion may have been created in the past through the use of a wide variety of other designations for these agents such as paraprobiotics, parapsychobiotics, ghost probiotics, zombie probiotics, metabiotics, tyndallized probiotics and bacterial lysates. Nevertheless, proponents of the term paraprobiotics have maintained that the definition of these as “non-viable microbial cells (either intact or broken), or crude cell extracts, which, when administered (orally or topically) in adequate amounts, confer a benefit on the human or animal consumer” is distinctive from that proposed for “postbiotics” (Taverniti and Guglielmetti, 2011; Siciliano et al., 2021). However, despite some strong support for retention of “paraprobiotic” as a distinctive term (Aguilar-Toalá et al., 2021) the ISAPP panel has more recently reaffirmed its recommendation for the unified application of the term postbiotic for all of these products (Salminen et al., 2021a; Vinderola et al., 2022).

#### Oral probiotics

Unlike most of the traditional intestinal tract-derived probiotics that are principally intended to provide gut-related health benefits, the oral probiotics are originally sourced from the oral microbiota. The beneficial outcomes of oral probiotics primarily (but not necessarily exclusively) result from their interaction with microbial and/or host cells within the oral cavity (Hale et al., 2016). This distinguishes *bona fide* oral probiotics from the more traditional “orally administered probiotics” (such as those present in yogurts or in products predominantly comprising intestinal lactobacilli and bifidobacteria) that in general do not persistently localize within the oral microbiota following their ingestion. Interestingly, many of the earlier endeavors to identify bacteria capable of fulfilling the functional role of oral probiotics had focused upon re-evaluating some of the already well-established intestinal probiotics, in spite of the inability of these strains to achieve any substantial persistent colonization within the oral microbiota.

## Bacteriocins, BLIS, RiPPs, and AMPs

**FIGURE 1:**
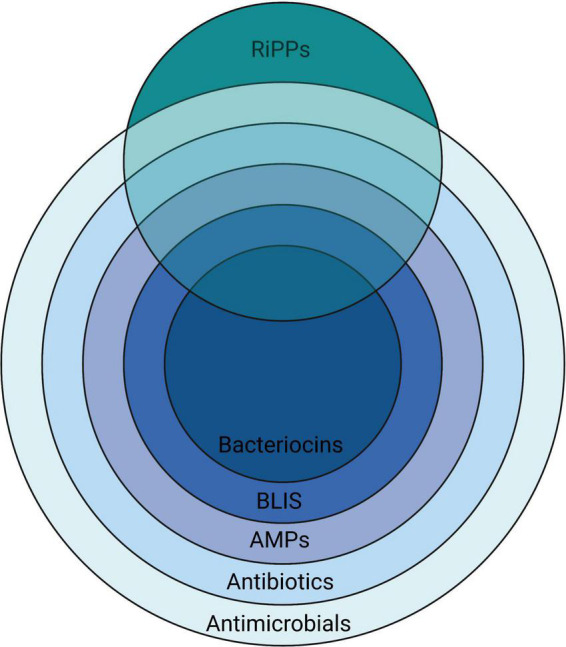
A nested overview of the contemporary usage of the terms used to describe various chemical agents having activity against microorganisms, illustrating how they relate to the more broadly applicable terms antibiotics (literally “opposing life”–used for any substance active against microbes, but in current usage referring to naturally-produced agents) and antimicrobials—a term that is also inclusive of synthetic substances. Created with BioRender.com.

A remarkably heterogeneous array of chemical entities have been shown to be inhibitory to the growth of microorganisms (Figure 1). The peptidic nature of some introduces a degree of specificity to their antimicrobial activity.

### Bacteriocins

The term bacteriocin was first used by Jacob et al. (1953) to categorize the then newly-discovered group of colicins and colicin-like bactericidal antibiotics produced by certain members of the *Enterobacteriaceae*. The typically plasmid-encoded synthesis of these bacteriocins was shown sometimes to be lethal to the producing cell in a process of altruistic behavior referred to as “programmed cell death” and their adsorption to sensitive cells was dependant on the presence of specific receptors. The bacteriocins were differentiated from most of the “classical” chemotherapeutic antibiotics that were also being discovered at around that time because of their proteinaceous composition and also the relatively narrow targeting of their anti-bacterial activity which typically was focused upon susceptible bacteria of species either the same as or closely related to that of the bacteriocin producer (Jacob et al., 1953).

### BLIS

This term was initially devised as an acronym for “Bacteriocin-Like Inhibitory Substances” and its intended application was as a preliminary descriptor for proteinaceous antibiotic activities produced by bacteria when tested *in vitro* against some closely related bacteria (Tagg, 1991, 1992). BLIS have been defined as “bacterial peptide or protein molecules, released extracellularly, that in low concentrations are able to kill certain other closely related bacteria by a mechanism against which the producer cell exhibits a degree of specific immunity”. This definition is not restrictive to substances having plasmid-borne genetic determinants. Nor does it imply a requirement for lethal biosynthesis, specific receptors or a particularly narrow activity spectrum, all of which were cornerstone elements of the original specifications for bacteriocins (Jacob et al., 1953).

### RiPPs

The ribosomally synthesized and post-translationally modified peptides (RiPPs) are a diverse class of natural products of ribosomal origin. Consisting now of more than 20 sub-classes, RiPPs are produced by prokaryotes, eukaryotes, and archaea, and some possess a wide range of alternative biological functions, besides anti-microbial activity. Some RiPPs such as the lantibiotics also classify as bacteriocins because of their demonstrable targeted bactericidal activity (Arnison et al., 2013; Li and Rebuffat, 2020).

### AMPs

The antimicrobial peptides (AMPs) are small, generally cationic, peptides from a wide variety of sources that can have relatively broad spectrum inhibitory activity against microorganisms (including bacteria, viruses, parasites, and fungi) by targeting membranes or specific intracellular components (Hale and Hancock, 2007; Omardien et al., 2016). The AMPs have been classified on the basis of their (1) source, (2) activity, (3) structural characteristics, and (4) amino acid-rich species (Huan et al., 2020).

## An overview of the bacteriocins of gram-positive bacteria

The first documentation of antagonistic interactions between bacteria has been attributed to Louis Pasteur. In his Florey (1946), commented that Pasteur and Joubert (1877) had reported that the growth of the anthrax Bacillus in urine was inhibited by the concomitant presence of “common bacteria” (probably *Escherichia coli*) and that this perhaps justified “the highest hopes for therapeutics” (translated). Subsequently, numerous attempts were made to control infections such as diphtheria and anthrax by dosing patients or vulnerable contacts with a variety of non-pathogenic antagonistic microorganisms. The mechanisms of these putative “microbial interference” activities were never fully understood (Florey, 1946). With the discovery of penicillin by Fleming in 1929 the antibiotic era was launched and interest quickly waned in the development of microbial interference as a strategy for infection prevention. The documented investigation of bacteriocins actually dates from 1925, when Gratia reported that filtrates from cultures of *Escherichia coli* V inhibited the growth of some other *E. coli* strains (Gratia, 1925). The active principle was later named “colicine” by Gratia and Fredericq (1946). Bacteriocinogenicity (the production of bacteriocins) has subsequently been characterized as an apparently essential survival characteristic for both Gram-negative and Gram-positive bacteria (Klaenhammer, 1988; Riley and Wertz, 2002). Similar molecules, named the archaeocins (Price and Shand, 2000), are also produced by members of the *Archaea*. For bacteriocin-producing bacteria there is inevitably a trade-off between the benefits to be obtained from their increased competitiveness versus the fitness cost incurred by the heightened metabolic load associated with the production of the bacteriocin molecules and also the mandatory homologous bacteriocin immunity (viz. protective shield) in the host cell (Blanchard et al., 2016). An important consideration is the relative benefit to the cell of constitutive bacteriocin production compared to quorum sensing controlled production. Interestingly it appears that in most natural ecosystems the cost to the producer bacterium of cell density-mediated control over constitutive bacteriocin production and the associated host cell specific bacteriocin immunity is the more energetically favorable option (Blanchard et al., 2016).

The apparently ubiquitous production of bacteriocins by all members of naturally-occurring populations of bacteria infers and indeed validates their significance as indispensable survival attributes for bacteria in highly competitive natural ecosystems and highlights their contribution to the maintenance of long-term population stability within microbial communities *via* the suppression of over-exuberant proliferation by other family members competing for the same ecological niche (García-Curiel et al., 2021; Heilbronner et al., 2021). From the perspective of the producer cell, bacteriocin production must be tightly regulated since this is an energetically expensive activity and also potentially autotoxic should bacteriocin levels exceed the specific bacteriocin immunity capabilities of the producer cell. Understanding the conditions triggering induction *in situ* is important in order to regulate/optimize bacteriocin expression in the host tissues. More recently, a growing awareness of the mammalian host cell signaling (modulating) activities of molecules previously characterized as bacteriocins has raised important questions about the underlying raison d’être for these molecules (Lin et al., 2019; Małaczewska et al., 2019). Is the modulation (beneficial or otherwise) of the human host’s physiology/immunity/brain function actually the primary evolutionary selection for the expression of these molecules by our indigenous microbiota? Perhaps the anti-competitor (bacteriocin) activity of some of these molecules is an auxiliary function, aiding the retention of bacterial populations capable of expressing these molecules at levels that are most appropriate for their primary human cell signaling functions?

Several bacteriocin typing schemas were developed in the pre-genomic era for use as epidemiological tools to help track the movements of populations of coliform bacteria (Hamon, 1961) and *Pseudomonas aeruginosa* (Gillies and Govan, 1966; Tagg and Mushin, 1971), particularly within hospital settings. These had as their basis the *in vitro* demonstration of characteristic patterns of bacteriocin-mediated inhibitory activity produced by the test strains against specified sets of “indicator” bacteria. Nisin (named for Group N inhibitory substance) (Mattick et al., 1947) was isolated from *Lactococcus lactis* (Rogers and Whittier, 1928; Whitehead, 1933) and soon became established as the progenitor and indeed still remains the flagship molecule of the lantibiotic class of bacteriocins–molecules containing distinctive lanthionine or beta methyl lanthionine-mediated intra-molecular cross-linkages (Guder et al., 2000). The practical development of nisin as a food preservative followed rapidly (Delves-Broughton et al., 1996) and its successful commercial application actually pre-dates Florey’s development of penicillin (Florey, 1946). Subsequently, nisin and nisin-based probiotics have been widely used to obtain clinical benefits in the treatment of a variety of oral and systemic diseases (Nguyen et al., 2020).

It was soon recognized that other Gram-positive bacteria and especially the so-called lactic acid bacteria (LAB) are prolific producers of bacteriocin-like inhibitory substances (viz. BLIS) (Tagg et al., 1976; Jack et al., 1995; Heng et al., 2007c). The genus *Streptococcus* became an early focus for studies of the LAB bacteriocins, and an initial impetus was the search for a streptococcal bacteriocin targeting that classical human pathogen, *Streptococcus pyogenes*, as part of a strategy to limit the occurrence of rheumatic fever in school aged children (Tagg and Dierksen, 2003). Perhaps anomalously, the first of the streptococcal bacteriocins to be definitively characterized was actually produced by a strain of *Streptococcus pyogenes*. This bacteriocin, streptococcin A-FF22 (SA-FF22), was shown to be a lantibiotic, very closely resembling nisin (Tagg and Wannamaker, 1978). Subsequently, a streptococcal “BLIS fingerprinting” scheme was devised (Tagg and Bannister, 1979), modeled upon the principles that had been previously developed for the bacteriocin typing of Gram-negative bacteria. BLIS fingerprinting had as its basis the *in vitro* detection of both the production of (P-typing) and sensitivity to (S-typing) streptococcal BLIS activities. Use of this methodology soon established that the production of BLIS activity within the streptococci was both frequent and heterogeneous (Nes et al., 2007; Tagg, 2009) and that isolates of *Streptococcus salivarius*, *Streptococcus uberis* and also of the mutans streptococcus cluster were particularly prolific BLIS producers (Tagg, 1992).

**FIGURE 2:**
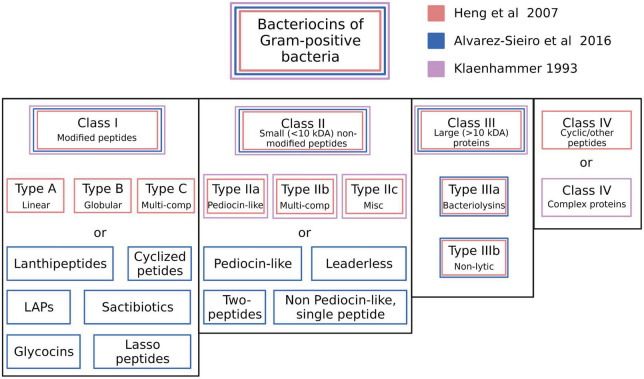
Schematic illustration of the bacteriocin typing schemes proposed by Klaenhammer (1993), Heng et al. (2007c), and Alvarez-Sieiro et al. (2016). The proposed classes for each scheme are color coded. Created with BioRender.com.

The recommendations for a classification framework for inhibitory agents conforming to the overarching characteristics of the bacteriocins continue to evolve as the depth of knowledge of their structural and biological characteristics and their genetic basis has increased. Klaenhammer (1993) initially attempted to introduce some order into the classification of the known bacteriocins of the LAB by proposing four major classes. Class I—post-translationally modified bacteriocins, such as nisin; Class II—small (<10 kDa) heat-stable membrane-active bacteriocins; Class III—larger (>30 kDa) heat-labile bacteriocins and Class IV—complex bacteriocins composed of essential lipid or carbohydrate moieties in addition to protein. Class II was further subdivided into IIa (anti-listerial peptides having the amino acid motif YGNGV/L in the N-terminal part of the peptide), IIb (two-component peptides) and IIc (thiol-activated peptides requiring reduced cysteine residues for activity) (Figure 2).

Subsequently, Heng et al. (2007c) proposed categorizing the then-known bacteriocins of Gram-positive bacteria according to a revised schema incorporating some of the cornerstone elements of the original Klaenhammer classification. Changes to the Klaenhammer scheme included: (1) elimination of class IV (the chemically complex bacteriocins) (2) Class III was subdivided into IIIa (bacteriolysins) and IIIb (non-lytic proteins) (3) the cyclic bacteriocins from Klaenhammer’s Class II now constituted a newly-defined Class IV (Figure 2). It was at around this time that the phenotypic BLIS fingerprinting of streptococcal isolates of diverse species and from a wide variety of sources was being used in the Tagg laboratory as a preliminary screening tool to facilitate the detection and differentiation of novel streptococcal bacteriocins. An outcome of this BLIS phenotype-driven strategy, the principles of which were essentially as more recently outlined by Twomey et al. (2021) was the *de novo* detection, purification and characterization of proteinaceous inhibitory molecules belonging to all four of the then-known classes of bacteriocins, as follows:

Class I (lantibiotics): SA-FF22 (Jack et al., 1994), streptin (Wescombe and Tagg, 2003), salivaricin A (Ross et al., 1993), salivaricin A1 (Johnson et al., 1979; Simpson et al., 1995; Tagg, 2004), salivaricins A2-A5 (Wescombe et al., 2006b), salivaricin B (Hyink et al., 2007), salivaricin G32 (Wescombe et al., 2012a), salivaricin 9 (Wescombe et al., 2011), salivaricin E (Walker et al., 2016), Smb (Hyink et al., 2005), BHT-A (Hyink et al., 2005), mutacin K8 (Robson et al., 2007) and nisin U (Wirawan et al., 2006). Subsequent studies have disclosed that transmissible megaplasmids present in some *Streptococcus salivarius* appear to be particularly adept at integrating (and expressing) lantibiotic loci (Wescombe et al., 2006a; Hyink et al., 2007; Barbour et al., 2020).

Class II (small (<10 kDa) non-modified peptides): mutacin N (Hale et al., 2004), mutacin IV and mutacin V (Hale et al., 2005), BHT-B (Hyink et al., 2005), streptocins STH_1_ and STH_2_ (Heng et al., 2007b), ubericin A (Heng et al., 2007a).

Class IIIa (large bacteriolytic proteins): zoocin A (Simmonds et al., 1997) and stellalysin (Heng et al., 2006b).

Class IIIb (large non-bacteriolytic proteins): dysgalacticin (Heng et al., 2006a,b), corynicin JK and enterococcin V583 (Swe et al., 2007) and streptococcin A-M57 (Heng et al., 2004).

Class IV (cyclic peptides): uberolysin (Wirawan et al., 2007).

The basic framework of the Heng bacteriocin classification scheme was subsequently adopted and adapted by several other research groups (Johnson et al., 2018; Ge et al., 2019). More recently however, the discovery of several structurally novel antimicrobial peptides produced by Gram-positive bacteria that also conform to the basic characteristics of the bacteriocins has prompted recommendations for further refinements to the classification of these molecules. For example, Alvarez-Sieiro et al. (2016) incorporated several additional subgroups within Klaenhammer’s Classes I and II, whilst also supporting the elimination of Klaenhammer’s Class IV (Figure 2). Their now-expanded Class I broadly comprised bacteriocin molecules that are ribosomally synthesized together with leader peptides which serve for enzyme recognition, transport, and for keeping the propeptide entities inactive throughout a variety of post-translational modifications that provide them with uncommon amino acids and structures (e.g., lanthionine, heterocycles, head-to-tail cyclization, glycosylations etc.). It should be noted that the Class I bacteriocins also cluster taxonomically within the rapidly expanding family of RiPPS (Arnison et al., 2013). Proposed subclasses within the Class I bacteriocin category were (i) lanthipeptides (Lagedroste et al., 2020), (ii) cyclized peptides (equivalent to Class IV of Heng et al. (2007c) (iii) LAPs, (iv) sactibiotics, (v) glycocins, and (vi) lasso peptides. Class II (the small unmodified bacteriocins) were subclassified as (i) pediocin-like (ii) two-peptide (iii) leaderless, and (iv) non-pediocin-like single peptides.

Subsequently, Acedo et al. (2018) and Zimina et al. (2020) have essentially concurred with the Alvarez-Sieiro et al. (2016) classification recommendations. In both cases retention of the >10 kDa Mr protein (i.e., Class III) category of bacteriocins was supported. On the other hand, Cotter et al. (2012) have argued for exclusion of the >10 kDa Mr proteins from classification as bacteriocins. It is our view however, that limiting the bacteriocins to molecules of mass <10 kDa would actually exclude most of the progenitor bacteriocins of Gram negative bacteria (viz the colicins) from classification as bacteriocins, as well as the growing collection of relatively large non-bacteriolytic proteinaceous bacteriocins, such as dysgalacticin (Heng et al., 2006a), streptococcin A-M57 (Heng et al., 2004) and the tailocins, now also shown to be produced by Gram positive bacteria (Acedo et al., 2018). The classical strategies employed for the screening, identification, purification, and characterization of bacteriocins and some recommendations for their refinement have been reviewed by Zou et al. (2018).

In summary, it is clear that microorganisms have evolved a broad repertoire of antimicrobial agents to support their survival, some of which conform to the cornerstone characteristics of the originally defined bacteriocin molecules *viz.* bacterial products having a bactericidal (“cin”) mode of action and with an underlying proteinaceous composition (i.e., they are primarily products of ribosomal synthesis). A number of apparent anomalies are evident, however. For example, the lantibiotic salivaricin A is bacteriostatic for most *S. pyogenes* and this is due to the widespread distribution of the Sal A immunity locus in *S. pyogenes* (Upton et al., 2001). Also, some of the so-called R23 bacteriocins produced by *Lactiplantibacillus plantarum* strain R23 have been found to have a bacteriostatic mode of action (Barbosa et al., 2021). In examples such as these there are undoubtedly strong host bacterium survival benefits linked to the modulated expression of the bacteriocins and/or bacteriocin immunity.

The bacteriocins, defined as such for our own convenience, of course represent only one component of an inevitably seamless continuum of bacterial antimicrobial activities. The abundance of whole genome sequence data expedites the detection and genetic analysis of putative bacteriocin clusters. With the advent and widespread application of genome mining technology, the knowledge base concerning the variety and distribution of bacteriocin-like determinants within the bacteria has flourished and a number of data base tools have been introduced to assist with the detection and cataloging of bacteriocin gene clusters (Sandiford, 2017; van Heel et al., 2018; Blin et al., 2021). Although the identification of putative new bacteriocins by database mining has been a promising innovation, the practical potential and the microbiome significance of these determinants is of course difficult to evaluate in the absence of suitable expression systems. The presence of bacteriocin, AMP and RiPP clusters can now be screened for using a variety of bioinformatic tools and databases, including antiSMASH 6.0 (Blin et al., 2021), BACTIBASE (Hammami et al., 2010), APD3–an antimicrobial peptide database (Wang et al., 2016, 2022), LABiocin–a database designed specifically for lactic acid bacterial bacteriocins (Kassaa et al., 2019) and BAGEL4—a web server available to mine RiPPs and bacteriocins (van Heel et al., 2018). InterProScan (Jones et al., 2014) can be used to assess the putative function of encoded proteins and identify protein domains and key sites (Javan et al., 2018). Fields et al. (2020) developed an *in vitro* pipeline for peptide design and testing using machine learning to predict 20-mer candidate peptides for synthesis followed by laboratory evaluation of their antimicrobial and cytotoxicity activities. Indeed, today, with the widespread availability of contemporary genome mining/annotation/cataloging tools, most putative bacteriocin loci are first detected not by classical phenotypic strain screening approaches, but in database formats prior to the subsequent confirmation of their phenotypic expression.

## Some practical applications of bacteriocins in health management

In recent years, the recognition of the relatively narrow activity spectra, high molecular stability and low antigenicity of many of the bacteriocins has led to increased interest within the scientific community in the potential for their development as novel therapeutic agents for infection control in humans and animals (O’Connor et al., 2020). Indeed, cognizance of the central role of the microbiota in human and animal health (Hernández-González et al., 2021) and a growing awareness of the fact that bacteriocins cause much less collateral damage to the host microbiome than classical antibiotics makes them increasingly a highly desirable therapeutic option (Desriac et al., 2010; Svetoch and Stern, 2010; Cotter et al., 2012; Upton et al., 2012; Meade et al., 2020; Soltani et al., 2021; Fernandes and Jobby, 2022). State of the art innovations are now under development to optimize the delivery of bacteriocin preparations (Radaic et al., 2020). The development of novel antibiotics is not keeping pace with resistance development, and this has led to a revival of practical interest in bacteriocins or bacteriotherapy for infection control. Also, it is now recognized that the application of personalized cocktail combinations of probiotics for infection control has considerable potential as a tool to help limit the emergence of antibiotic resistance (Hols et al., 2019). Simons et al. (2020) have provided a comprehensive overview of the bacteriocins of Gram positive and Gram negative bacteria and a commentary about their potential importance in the search for agents having activity against multi-drug resistant bacteria. Apart from their potent antibacterial activity [with minimum inhibitory concentrations (MICs) often in the nanomolar range], some bacteriocins have also been shown to have direct antiviral activity (Tiwari et al., 2020; Todorov et al., 2021).

It is being increasingly recognized that a number of the molecules characterized as “bacteriocins” because of their documented ability to suppress the *in vitro* growth of prokaryote competitor bacteria may also be adept at physically and functionally interacting with eukaryotic cells of the host–potentially prompting their applications for the treatment of cancers (Kaur and Kaur, 2015; Rodrigues et al., 2019; Sadri et al., 2022) or for modulating neuronal cell activity (Bowland and Weyrich, 2022). Notably certain lantibiotics have been shown to exert beneficial therapeutic and immunomodulatory effects (van Staden et al., 2021) and recently, both salivaricin A2 and salivaricin B (the two key lantibiotic products of *S. salivarius* BLIS K12), have been shown to reduce disease severity in an animal model of rheumatoid arthritis (Li X. et al., 2022). Indeed, it is evident that some of these molecules may perform key functions for the human host relating more to their specific eukaryotic cell interactions than to their anti-prokaryotic cell activities. Understanding and purposefully manipulating these bacteriocin-mediated activities now provides an innovative contemporary strategy for medicine.

## The influence of probiotic-mediated BLIS activity on the composition and functionality of the oral microbiome

In the past, bacteriocin production has largely been considered as a desirable rather than a key probiotic trait (Hegarty et al., 2016). The prospering and indeed the very survival of a bacterium within highly competitive microbial populations is significantly influenced by the controlled expression of its repertoire of bacteriocin determinants. It is evident however, that bacteriocins will only provide an advantage if (a) the benefit of their anti-competitor activity exceeds the metabolic cost of their production, (b) key mutualistic partner bacteria are not targeted and (c) any major competitor bacteria within that ecosystem cannot develop bacteriocin resistance (Heilbronner et al., 2021). It is recognized that bacteriocins are only one component of the biotoxic arsenal of microorganisms. Barbour et al. (2022) have recently reviewed current knowledge of the contribution of a wide variety of microbial metabolites to a microbe’s life and death within the oral microbiome and the implications of this (both good and bad) for the human host.

The expression of bacteriocins and of the homologous specific bacteriocin immunity by a bacterium is an energy demanding process requiring the coordinated action of multiple gene products. The high cost to the cell of bacteriocin activity infers that bacteriocinogenicity city must confer a significant survival benefit upon the host bacterium. It is evident that the proportion of bacteriocin-producing *S. salivarius* is very high and in most cases the genetic determinants for their sometimes multiple bacteriocins appear to be localized to (>100 kbp) transmissible megaplasmids (Wescombe et al., 2006a,^2010^). *S. salivarius* megaplasmids seem to serve as genetic receptacles for the acquisition and expression of a heterogeneous array of bacteriocin loci (Walker et al., 2016), putatively gathered from a variety of other oral streptococci.

The type-A lantibiotic salivaricin A produced by the *S. salivarius* probiotics BLIS K12 and BLIS M18 functions by forming pores in the cytoplasmic membranes of target (competitor?) bacteria (Geng et al., 2018). Pore formation, however, only occurs if there is a sufficiently high potential difference across the target cell membrane (Wescombe et al., 2009). On the other hand, salivaricin B (produced by BLIS K12, but not by BLIS M18) interferes with cell wall biosynthesis and specifically with septum formation. It does not appear to penetrate the cytoplasmic membrane (Barbour et al., 2016). These observations support the hypothesis that these lantibiotics may help modulate the oral microbiota composition by killing relatively rapidly multiplying competitor bacteria—such as, for example, pathogenic *S. pyogenes* during the acute stage of a streptococcal throat infection. Interestingly, with the notable exception of serotype M4 strains, it appears that most *S. pyogenes* harbor and express salivaricin A immunity determinants (viz SalY- a membrane-spanning region of a putative efflux pump) which may provide these *S. pyogenes* with cross protection against eukaryotic cationic peptides (Phelps and Neely, 2007). Another consequence of this is that salivaricin A is bacteriostatic for these *S. pyogenes* (Upton et al., 2001). Perhaps this represents a satisfactory evolutionary arrangement for both *S. pyogenes* and its human host? In an oral microbiome permeated by salivaricin A these *S. pyogenes* will be relatively constrained in their replication (as in a non-disease-causing carriage state)—but nevertheless some cells will survive! It is evident and indeed should be anticipated that pathogenic bacteria including the opportunistic beta hemolytic streptococci (Vogel and Spellerberg, 2021) are also equipped with their own bacteriocin armory and so the outcome in terms of clonal survival will inevitably also be influenced by a variety of additional factors including the relative cell numbers, adhesion avidity, nutritional requirements and host immune defenses etc.

Bacteriocin production is strongly influenced by both genetic and environmental factors and in order to conserve energy, bacteriocin expression can temporarily be turned off (Wescombe et al., 2006b). Alternatively, bacteriocin genetic loci may be either inactivated (*via* mutation) or completely lost (as happens in *S. salivarius* upon megaplasmid elimination) should that bacteriocin fail to confer a survival advantage to the host bacterium. Some bacteriocins inhibit at high concentrations, but have a signaling function when present in low concentrations (Fajardo et al., 2008). Bacteriocins produced by probiotic strains can act as quorum sensing molecules or auto-inducing peptides (Mignolet et al., 2018, 2019; Hols et al., 2019). The targeted killing capability of bacteriocins produced *in situ* by probiotic bacteria can potentially serve to confer a very substantial anti-infection benefit to the host.

Most probiotic bacteria currently under development are now routinely examined for their expression of bacteriocins targeting microbes that are potentially either pathogenic for the human host or that may contribute to the formation of dysbiotic populations within the host’s indigenous microbiota. The importance of bacteriocinogenicity as a keystone probiotic trait is now well-established (Hegarty et al., 2016). It should be noted however, that the exhibiting of bacteriocin-mediated antagonism *in vitro* by a probiotic candidate strain does not necessarily correlate with competitiveness *in vivo* as demonstrated in mouse model studies by Culp et al. (2022).

In summary, bacteriocin production may facilitate: (i) the *de novo* introduction of the bacteriocin producer bacterium within a pre-established microbiome (ii) resisting the invasion of a niche colonized by the bacteriocin producer by other competitor bacteria, including potential pathogens (iii) beneficial modulation of the host immune system (Dobson et al., 2012).

## Desirable characteristics of an oral probiotic

Initially, many of the candidate probiotics examined for their potential to produce health benefits in the oral cavity were of intestinal origin. Pragmatically, in some cases, this may have been because these strains had already satisfied the prerequisite safety and regulatory hurdles. However, intuitively it seems that when seeking a probiotic for efficacious application in the oral cavity, a specialist oral cavity bacterium may be anticipated to be best equipped to do the job, since they have been evolutionarily conditioned for attachment and proliferation within an oral niche and their repertoire of anti-competitor molecules seems more likely to be focused against other microbes that are aggressive oral microbiome insurgents.

**FIGURE 3:**
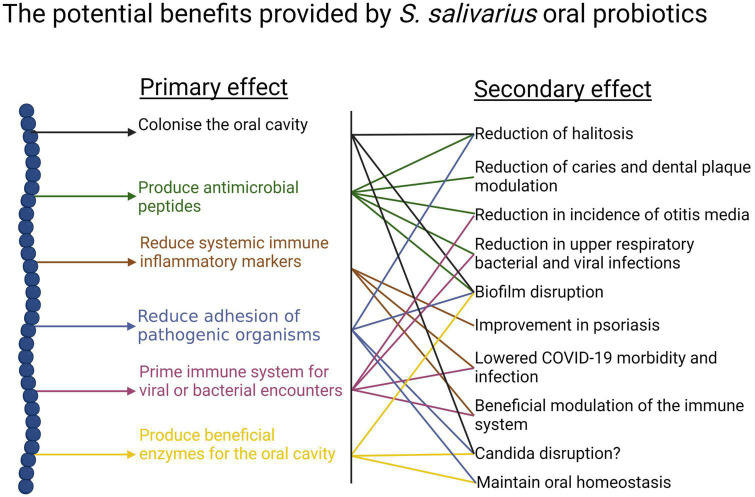
Beneficial activities (primary effects) and outcomes (secondary effects) within the human oral cavity and beyond that have been associated with the use of the *S. salivarius* probiotics BLIS K12 and BLIS M18. Created with BioRender.com.

**FIGURE 4:**
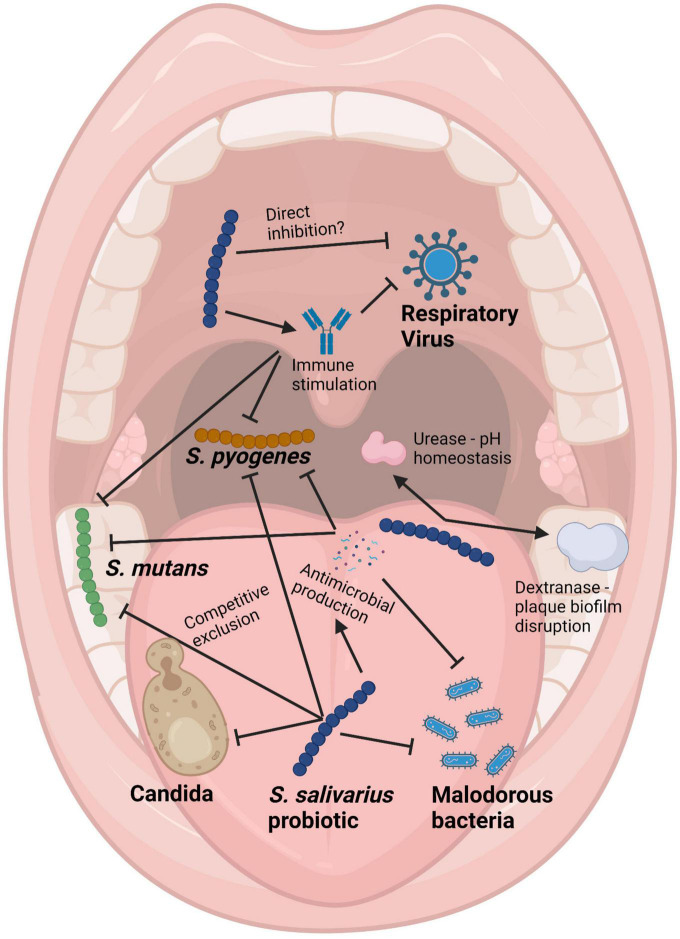
Schematic of the mouth showing some of the health benefits to the host provided by *Streptococcus salivarius* probiotics. Pointed arrows represent production of molecules, while blunt arrows signify inhibition. Created with BioRender.com.

The established requirements for efficacious oral probiotics such as viability, adherence ability, provision of health benefits, safety, and delivery efficiency mirror the established requirements for intestinal probiotics (Papadimitriou et al., 2015; How and Yeo, 2021). Additional key attributes for an oral cavity probiotic for use in humans include:

(i) Anti-competitor (*viz* anti-potential oral cavity pathogen) activity (Figures 3, 4).

(ii) High natural oral cavity prevalence of the probiotic species in healthy humans.
(iii) Long term persistence in the oral cavity.

It is difficult for probiotic cells seeded into the human host in a planktonic state to integrate within a pre-established microbial community. Because of this, some key characteristics of an effective oral probiotic will include strong anti-competitor (e.g., bacteriocin or BLIS) repertoires as well as surface structures facilitating their specific adhesion within the oral cavity (Figures 3, 4). Other keystone attributes include an ability to beneficially modulate the functionality of human host cells, potentially protecting the host against inflammation/apoptosis induced by pathogens or in the promotion of homeostasis (Cosseau et al., 2008; Guglielmetti et al., 2010) and the release of enzymes beneficial for the host such as urease (countering acidogenesis) (Chen et al., 2000) and dextranase (Walker et al., 2016) (reducing plaque accumulation) (Figures 3, 4).

Scientific and public interest in the potential for further applications of probiotics as medical therapies is now steadily increasing. Progress in the past has been constrained by:

(i) Lack of standardization, making it difficult to compare and replicate results from different studies.
(ii) Limited understanding of mechanisms of action in providing health benefits, making it difficult to design targeted therapies and predict outcomes.
(iii) Inadequate clinical evidence due to small sample sizes, heterogeneous study populations and inconsistent outcomes.
(iv) Regulatory hurdles. Since probiotics are considered to be dietary supplements rather than drugs it is more difficult to ensure the quality and safety of probiotic products.

## Pioneer applications of streptococci as oral probiotics

Many of the microbes consistently found to inhabit the human oral cavity have specifically evolved to operate optimally only within their human host and amongst the more than 700 species already identified, the streptococci are numerically prominent. Following birth, members of the species *Streptococcus salivarius* are amongst the first to inhabit the oral cavity and they play an important role in the underlying assembly of the oral microbiota (Abranches et al., 2018). The basis for selection of streptococcal strains for potential use as oral probiotics in so-called “bacteriotherapy” (Bellussi et al., 2019) or “replacement therapy” (Tagg and Dierksen, 2003) was not informed by in depth knowledge of the molecular basis for the *in vitro* observations of their anti-competitor activity against potentially pathogenic microbes. The underlying objective behind these oral microbiota modulation endeavors was to achieve implantation and persistence within the normal microbiota of relatively innocuous “effector” bacteria that could either competitively exclude or at least prevent the outgrowth of potentially disease-causing bacteria, without significantly disturbing the balance of the existing microbial ecosystem.

The principal target in the earliest applications of streptococcal probiotics was that classical human pathogen, *S. pyogenes*. Sanders et al. (1977) speculated that the relatively reduced occurrence of *S. pyogenes* pharyngitis episodes in adults by comparison to children may be attributable to adults having larger pharyngeal populations of non-hemolytic streptococci that were capable of producing bactericidal activity against *S. pyogenes*. Later, these researchers described a low molecular weight pantothenic acid antagonist that they named enocin as a *S. salivarius* product potentially contributing to anti-*S. pyogenes* activity (Sanders and Sanders, 1982). Meanwhile, Sprunt and Leidy (1988) demonstrated that a single inoculation with alpha hemolytic streptococcus strain 215 appeared to help beneficially restore the “protective balance” of the nasopharyngeal microbiota in infants. Subsequently, Grahn and Holm (1983) reported that the prevalence of oral alpha-hemolytic streptococci inhibitory *in vitro* to *S. pyogenes* was lower in children who became infected during a community outbreak of streptococcal tonsillitis. In follow-up studies these researchers showed that the use of an oral spray containing four strains of alpha hemolytic streptococci following a preliminary course of antibiotics was effective in reducing subsequent tonsillitis episodes in children (Roos et al., 1993, 1996; Falck et al., 1999). One of the inhibitory strains, the colicin V-encoding *Streptococcus oralis* 89a (Sidjabat et al., 2016), has subsequently also been co-administered with *S. salivarius* 24SMB in an on-going series of probiotic studies (see later). Prominent among the pioneering studies of use of *S. salivarius* to reduce tooth decay was the rough-colony forming (on sucrose-containing medium) strain TOVE-R. TOVE-R was found to competitively displace *S. mutans* and *S. sobrinus* from the teeth of rats and thereby inhibit tooth decay (Tanzer et al., 1985a,b). In none of these pioneering studies was the identity of the anti-streptococcal activity clearly defined.

## BLIS-producing *Streptococcus salivarius* as oral probiotics for humans

*Streptococcus salivarius* is perhaps the most innocuous of all the bacterial species known to colonize the human oral and nasopharyngeal mucosae in large numbers. Infants typically acquire the mother’s predominant strain of *S. salivarius* within hours of birth (Carlsson et al., 1970; Tagg et al., 1983). *S. salivarius* is present at levels of up to 1 × 10^7^ colony forming units (cfu) per milliliter of saliva and based on the daily estimated average consumption of saliva in adults (up to 1.5 liters), large numbers (estimates of 1 × 10^10^ cfu) are ingested daily. Within the oral microbiota, *S. salivarius* has been shown to be especially abundant on the dorsum of the tongue (Mark Welch et al., 2019). *S. salivarius* is also present in breast milk, an important source of the bacterium in the early months of life (Tagg et al., 1983) and it is also a significant and apparently influential member of the intestinal microbiota (Hols et al., 2019). A recent case control microbiome study has indicated that *S. salivarius* populations (a) are relatively over-represented in the nasopharyngeal microbiotas of healthy subjects and (b) can demonstrate *in vitro* inhibitory activity against respiratory pathobionts (Jörissen et al., 2021). Depletion of *S. salivarius* populations can lead to unbalanced “dysbiotic” overgrowth of the potentially harmful *Candida* spp. and various black-pigmented anaerobes, manifesting clinically as oral thrush (Liljemark and Gibbons, 1973) and halitosis (Kazor et al., 2003), respectively. It has been speculated that due to its strategic intra-oral cavity location, versatile and abundant anti-competitor weaponry and its substantial numbers, *S. salivarius* may have an important population surveillance and management (i.e., “sentinel”) role within the oral microbiota (Tagg and Dierksen, 2003). A recent microbiome analysis of children susceptible to chronic otitis media with effusion has also pointed to the important role of *S. salivarius* as a health-associated and prevalent inhabitant of the human nasopharynx (Jörissen et al., 2021).

*S. pyogenes* infections in school aged children and their major sequel rheumatic fever was the primary focus of our own pioneering search for an oral probiotic. Seeding of the oral microbiota with a probiotic having anti-*S. pyogenes* activity was reasoned to potentially provide a means of reducing the occurrence of streptococcal pharyngitis and also an alternative to the currently mandatory antibiotic prophylaxis regimens for the prevention of rheumatic fever recurrences. In the absence of an effective vaccine, *S. pyogenes* infections and their sequelae continue to cause widespread illness and mortality. Not all children appear equally susceptible to *S. pyogenes* infection. Our underlying hypothesis was that children experiencing fewer acquisitions of *S. pyogenes* were naturally protected due to their harboring of indigenous oral cavity populations of bacteria producing anti-*S. pyogenes* BLIS activity (Tagg, 2009). Our preliminary studies (Tagg and Russell, 1981; Dempster and Tagg, 1982) had indicated that *S. pyogenes* was particularly susceptible *in vitro* to an apparently wide variety of the BLIS activities produced by the human oral cavity commensal species *S. salivarius*. These observations encouraged us to conduct a 6-years longitudinal study of one hundred and three 5–6 years old Dunedin children, monitoring the occurrence of *S. pyogenes* pharyngitis episodes and their salivary content of BLIS-producing *S. salivarius* (Tagg et al., 1990). Throat cultures were plated on blood agar (to detect *S. pyogenes*) and saliva samples on Mitis Salivarius agar were screened for colonies of oral streptococci producing *anti-S. pyogenes* BLIS activity in simultaneous and deferred antagonism tests. These studies demonstrated that children experiencing relatively fewer *S. pyogenes* infections often harbored populations of *S. salivarius* displaying a strong (P-type 677) spectrum of inhibitory activity when tested *in vitro* using the Tagg and Bannister (1979) BLIS “fingerprinting” assay. The designation P-type 677 equates to inhibition of eight of the nine standard indicator bacteria used in the “fingerprinting” protocol.

**FIGURE 5:**
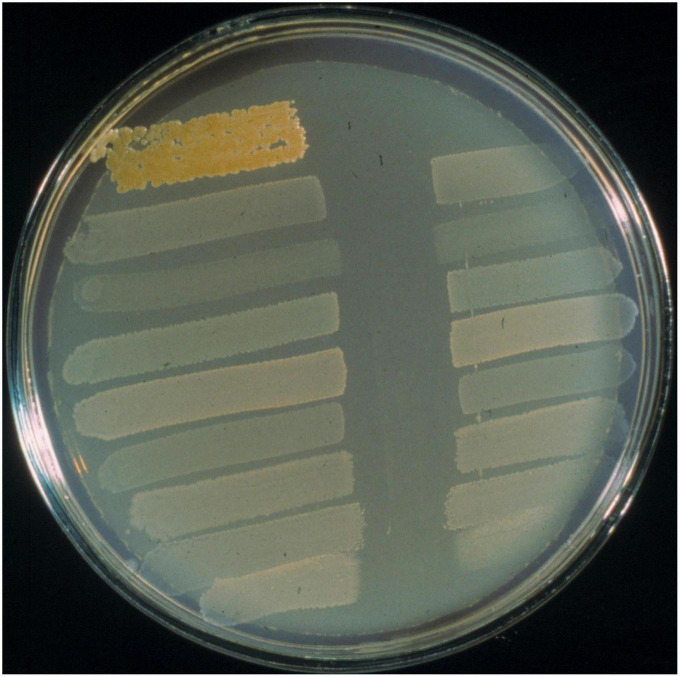
BLIS “fingerprinting” assay of *S. salivarius* BLIS K12 using the deferred antagonism test methodology as described by Tagg and Bannister (1979). Interference with the growth of all nine standard indicators (as shown here) is referred to as BLIS P-type 777 activity.

The P-type 677 *S. salivarius* appear typically to be producers of the lantibiotic salivaricin A (Ross et al., 1993). Attention however, soon became focused upon one child in the study who for more than 12 months had retained a predominant oral *S. salivarius* population producing especially strong (P-type 777) (Figure 5) BLIS activity (i.e., inhibitory to all nine of the standard indicator bacteria), during which period they had experienced no new *S. pyogenes* infections. *S. salivarius* strain K12 (*viz* BLIS K12), selected as the prototype of these P-type 777 isolates, was shown to produce the lantibiotic salivaricin B in addition to salivaricin A. In a follow-up 10 months study of 780 school children 11% had predominant P-type 226 *S. salivarius* populations, 9% yielded P-type 677 isolates (i.e., putative salivaricin A producers) and around 20% of the children had *S. salivarius* of various other P-type designations. The group of children harboring P-type 677 *S. salivarius* populations experienced significantly fewer *S. pyogenes* infections than did the other children in the study (Dierksen and Tagg, 2000). Meanwhile, a survey of saliva samples from 180 young adults showed that 43% had BLIS-producing *S. salivarius* of 13 different P-type designations. Of these subjects, 19 (10.5%) yielded P-type 677 *S. salivarius* and no P-type 777 isolates were detected (Tagg et al., 1983). Examination of 14 independently-sourced *S. salivarius* producing especially strong (P-type 777) BLIS activity showed that they all harbored mega-plasmids, nine of which encoded various combinations of the lantibiotics salivaricin A, salivaricin B, and streptin (Wescombe et al., 2006a). Megaplasmids appear to be a major asset for *S. salivarius* and are clearly important for the assembly of their extensive and versatile BLIS portfolios.

*Streptococcus salivarius* BLIS K12 was initially focused upon as the preferred candidate for development as an oral cavity probiotic because of its especially strong *in vitro* anti-*S. pyogenes* activity and its apparently safe and protective long-term persistence within the oral microbiota of its original source human subject (Burton et al., 2011). Extensive safety studies affirmed its “generally recognized as safe” (or “GRAS”) with no objection in the USA and in 2001 *S. salivarius* BLIS K12™ was distributed commercially by the New Zealand company BLIS Technologies Ltd (Dunedin, New Zealand)^1^ as the international prototype of the BLIS-producing oral probiotics. BLIS K12 also (a) binds with strong avidity to human epithelial cells *in vitro* and prevents attachment of *S. pyogenes* (Fiedler et al., 2013) (b) persists following ingestion in the human oral cavity (Horz et al., 2007) (c) reduces *in vitro* biofilm formation by *S. mutans* and several other oral pathogens (Stašková et al., 2021) and reduces the immune activation induced by periodontal disease pathogens (MacDonald et al., 2021). Subsequently BLIS K12 has been found to effect a broad spectrum of health benefits (Figures 3, 4) including the reduction of upper respiratory tract bacterial and viral infections in children (Di Pierro et al., 2014) and adults (Di Pierro et al., 2013), alleviation of halitosis (Burton et al., 2006), reduction of secretory otitis media (Di Pierro et al., 2015a) and *Candida albicans* infections (Ishijima et al., 2012), beneficial modulation of the immune system (Cosseau et al., 2008; Laws et al., 2021, 2022), stimulation of antiviral immune defenses (Bouwer et al., 2013) and the countering of a wide variety of pathologies that are linked to oral microbiota dysbiosis. The reader is referred to Supplementary Table A which documents the >60 publications focusing on probiotic applications of *S. salivarius* BLIS K12.

From the perspective of assisting with the maintenance of good oral health however, one apparent significant deficiency in the antimicrobial spectrum of BLIS K12 was the mutans streptococci, recognized as the principal etiological agents of dental caries. Our initial endeavors to identify a streptococcus having strong *in vitro* BLIS activity against mutans streptococci (James and Tagg, 1988) pinpointed *Streptococcus zooepidemicus* 4881, a producer of the potent Class IIIa bacteriocin zoocin A (Simmonds et al., 1997). This strain however, had low potential for oral probiotic development since the species has some disease associations and it cannot be considered to be an oral cavity commensal. A more ecologically compatible probiotic candidate strain given preliminary consideration was *Streptococcus sanguinis* K11 (Skilton and Tagg, 1992). Unfortunately however, the BLIS activity of strain K11 (streptococcin san-K11) appeared limited to the mutans streptococcus species *Streptococcus rattus*. At this time the strongest *S. salivarius* producer of *in vitro* anti-mutans BLIS activity was the P-type 777 strain Min5, but unfortunately its activity seemed limited to the mutans streptococcus species *Streptococcus cricetus* (James and Tagg, 1988).

Some *S. salivarius* isolates have been shown to release into human saliva quantities of the enzymes urease (Chen et al., 2000; Burton et al., 2013) and dextranase (Igarashi et al., 2001; Walker et al., 2016), which may potentially modulate dental plaque acidification and accumulation, respectively. In view of this it was decided to also incorporate evaluation of these enzyme activities as components the phenotypic screen of this laboratories BLIS-producing *S. salivarius* for an appropriate anti-mutans probiotic candidate. Some strains, such as *S. salivarius* JH (Walker et al., 2016), although exhibiting very potent anti-mutans BLIS activity had to be dismissed from further consideration due to their concomitant production of hemolytic activity—a characteristic of some *S. salivarius* (Tompkins and Tagg, 1989).

Ultimately, *S. salivarius* BLIS M18™ was focused upon for commercial development due to its unusually strong (for *S. salivarius*) activity against the mutans streptococcus species *S. mutans* and *S. sobrinus*, in addition to being a strong producer of both urease and dextranase enzyme activities. Four bacteriocin loci have been identified in the BLIS M18 genome: salivaricin A2, salivaricin 9, and salivaricin MPS (all megaplasmid-encoded) and the chromosomally-encoded lantibiotic salivaricin M which appears to be responsible for much of the observed anti-mutans streptococcal activity of this bacterium (Heng et al., 2011; Wescombe et al., 2012b). The range of specific probiotic applications of BLIS M18 have largely been prompted by idiosyncrasies of its BLIS spectrum (Supplementary Table B). Successful outcomes have been reported not only for its impact on Cariogram index (Di Pierro et al., 2015b; Tandelilin et al., 2018; Poorni et al., 2022), reduction of gingivitis/periodontitis (Scariya et al., 2015) and halitosis (Benic et al., 2019; Yoo et al., 2020) and modulation of black staining plaque (Gobbi et al., 2020), but also for its unanticipated postbiotic activity against colon cancer cells (Karaçam and Tunçer, 2022) and the important Gram-negative pathogens *P. aeruginosa* and *Klebsiella pneumoniae* (Tunçer and Karaçam, 2020).

**FIGURE 6:**
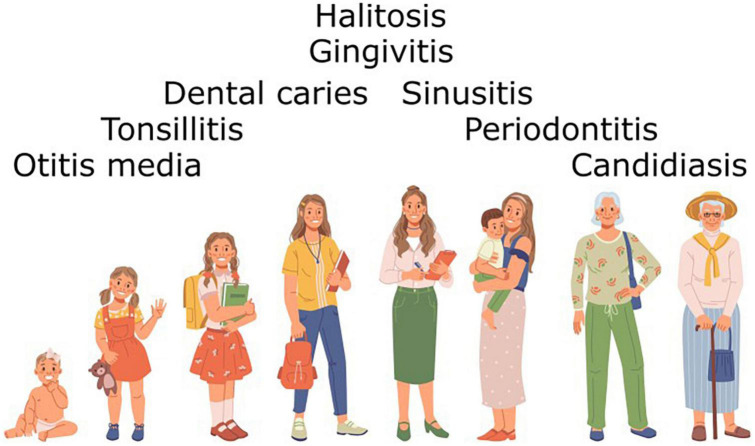
Diseases presenting at different stages of the life of the human host that have already been shown to be beneficially modulated by administration of BLIS-producing *S. salivarius* probiotics.

It should be noted that in the case of both BLIS K12 and BLIS M18 the primary criterion in the process of strain selection was the *in vitro* detection of a *S. salivarius* producing novel BLIS activities against potentially pathogenic or dysbiosis-associated target species within the oral and nasopharyngeal microbiotas. Subsequently however, the spectrum of microbial dysbiosis and pathologies of the human host that have been beneficially modulated through the use of these BLIS-producing *S. salivarius* probiotics has expanded to include the complete life-span of the human host (Figure 6). Although a steadily increasing number of *S. salivarius* isolates are now being touted as potential probiotic candidates, very few have as yet progressed for evaluation in human trials (Supplementary Table C). Indeed, the only other *S. salivarius* to have as yet been widely applied as a probiotic is *S. salivarius* 24SMB, more recently in combination with *S. oralis* 89a in nasal spray formulations (Marchisio et al., 2015; Manti et al., 2020). Unlike strains BLIS K12 and BLIS M18, *S. salivarius* 24SMB is megaplasmid-negative and does not encode any lantibiotic activities (Vertillo Aluisio et al., 2022). It does however, appear to encode a novel *blpU*-*K* bacteriocin activity and two class-IIb bacteriocins. The companion probiotic, *S. oralis* 89a, on the other hand is a producer of colicin V, so called originally because of the association of the ColV plasmid with increased virulence in *Escherichia coli* strains (Smith and Huggins, 1976) and possibly accounting for the observed relatively strong activity of this strain against Gram negative bacteria (Sidjabat et al., 2016).

Several strains of streptococcal species other than *S. salivarius* have either been marketed as probiotics or have been promoted as potential probiotics (Supplementary Table D). Foremost amongst these is the commercial oral probiotic product Probiora 3, which comprises a mixture of *S. rattus* JH145, *S. oralis* KJ3sm, and *Streptococcus uberis* KJ2sm (Zahradnik et al., 2009). Other streptococcal species that have been promoted for potential probiotic development include *S. cristatus*, *S. dentisani*, *S. sanguinis*, *S. parasanguinis*, *S. gordonii*, *S. mitis*, *S. oligofermentans, S. symci, Streptococcus* strain C17T, and *Streptococcus* sp. A12. Indeed, a steadily-increasing assemblage of streptococcal isolates from the human oral microbiota and various other sources have either already been given consideration or are currently under development for use as oral probiotics (Hale et al., 2016).

## Future prospects for oral probiotics

Much human illness is linked either directly (e.g., dental caries, periodontal disease, candidosis) or indirectly to the development of oral microbiota disequilibria (Figure 6). Not only can this contribute directly to the development of dental caries, periodontal disease and candidosis, but it may also facilitate initiation of the transition between carriage state and active infection by bacteria such as *S. pyogene*s as well as possibly influencing the onset of a wide variety of non-transmissible diseases. Indeed a surfeit of a number of periodontal pathogens has been identified as risk factors for an increasing repertoire of systemic diseases (Bourgeois et al., 2019). Consequently, research into the scientifically orchestrated beneficial applications of oral probiotics has received a substantial fillip as the evidence base underpinning the importance of establishing and maintaining a health-promoting oral microbiota continues to strengthen. Future research directions potentially facilitating the more widespread practical application of oral probiotics to the management and treatment of diseases of the human oral cavity and beyond include:

(i) Identification of optimal strains, dosage levels and delivery mechanisms.
(ii) Investigation of long-term safety.
(iii) Conducting of large-scale, well-controlled clinical trials.
(iv) Further development of regulatory guidelines.

With the advent of the antibiotic era, the further development of bacterial interference-based strategies for infection control was largely put “on hold.” More recently however, the efficacy of many therapeutic antibiotics has diminished due to the wide-spread propagation of antibiotic resistance. Combined with that there has been an associated resurgence of many infectious diseases and an increase in the numbers of immunologically compromised and aged human hosts. It is within this disturbing setting that probiotics are now increasingly being reconsidered either as an alternative or as a supplement to existing chemotherapies. With increasing interest now in probiotic interventions for the specific modulation of microbial dysbiosis in a wide variety of body tissues, attention is being focused upon the “mining” of the predominant microbes that are indigenous to these tissues as a prime source of potential probiotics that are optimally equipped to colonize and aid re-equilibration of the populations of microbes incumbent to these tissues.

Recently there has been renewed interest in understanding microbial behavior in natural habitats. Many chronic diseases can have complex etiologies (sometimes not conforming to classic Koch postulates) and there is mounting evidence for microbial interventions in a plethora of pathologies in the human host (Thomas et al., 2021), many of which are potentially either instigated or sustained by dysbiosis within the oral microbiome (Le Bars et al., 2017; Tiwari et al., 2020; Maitre et al., 2021b; Nguyen et al., 2021; Todorov et al., 2021; Gao et al., 2022; Rismayuddin et al., 2022; Sankarapandian et al., 2022). We all harbor personalized microbiomes based within our oral cavity biofilms, the balanced operations of which are a critical factor in our health status within the mouth -and beyond. The oral microbiome also has a “dark side” however, capable of eliciting disease—both oral and systemic. Ecological shifts within the microbiome may allow pathogenic microbes to elicit disease either due to their reinvigorated multiplication following emergence from a carriage status within the microbiome or due to their relatively unchallenged uptake following *de novo* entry into the oral cavity. As more detailed information becomes available concerning the population composition and dynamics of the oral microbiome and its genetic complement, this will lead to the informed development of more effective therapeutic and diagnostic approaches (Gao et al., 2018). Ultimately and inevitably, this should then contribute to the development of personalized health management programmes for the entire human/microbiome entity–or super organism. Already there is recognition of the potential for oral microbiome analysis in early life to identify possible microbial harbingers of future disease development in children followed by the administration of tailor made blends of specific oral probiotic “combos,” the composition of which can be adjusted in response to periodic monitoring of the composition of the host’s microbiome (Wescombe et al., 2009; Xiao et al., 2020).

The oral microbiome functions as a critical checkpoint for microbial visitors—harmless or harmful. Will refugee microbes gain acceptance and be assimilated within the oral microbiome or will they pass through for subsequent fitness checks in other parts of the body? Sophisticated profiling of the oral microbiome may indicate carriage of hibernating predators that are potentially capable of becoming a source of infection for the host or their close contacts. Alternatively, this profiling exercise may indicate levels of particular microbes that are potentially capable of adversely influencing host physiology. Indeed, both gingivitis and periodontitis have been linked to a two-way effect between oral and systemic disease (Bui et al., 2019). Furthermore, while the literature on the gut-brain axis is rapidly growing there is now also an undercurrent of scientific probing into the influence of oral microbes on brain function (Maitre et al., 2021a; Narengaowa et al., 2021). Recently, for example Ahrens et al. (2022) pointed to a potential association between an oral microbiome deficiency of the species *Alloprevotella rava* and suicidal ideation in young adults.

*Streptococcus salivarius* probiotics have already found successful application to the beneficial modulation of a wide spectrum of microbial dysbiosis impacting on human health. The principal oral bacterial infections currently identified and successfully targeted for probiotic intervention have been streptococcal pharyngitis, otitis media, halitosis and dental caries (Figure 6; Tagg, 2016; Hoare et al., 2017; Bustamante et al., 2020). Perhaps the time is now ripe for the accelerated development and practical application of BLIS-producing *S. salivarius* probiotics to the biotherapeutic control of other diseases and maladies of complex etiologies resulting from more subtle disturbances within the indigenous microbiota (Tagg, 2016). The targeted probiotic approach to microbiome management is cost effective, easily implemented and does not present many of the complications associated with immunization (hypersensitivity or antigenic cross-reactivity), chemotherapy (resistance development and direct toxicity) or other medications. The considerable scope for BLIS-producing *S. salivarius* to be beneficially utilized throughout the life of their natural human host perhaps encourages consideration of them as the prototype oral probiotics for all ages.

## Author contributions

JT conceived the scope of the manuscript and wrote the first draft. LH designed and prepared the figures, and assisted with the compilation of references. RJ assisted with the text editing and structure. JH advised on text content and flow. All authors contributed to the article and approved the submitted version.

## Footnotes

1
www.blis.co.nz
