# Supplementary material for: Beneficial modulation of human health in the oral cavity and beyond using bacteriocin-like inhibitory substance-producing streptococcal probiotics
Source: Front Microbiol. 2023 Mar 28;14:1161155. doi: 10.3389/fmicb.2023.1161155 (PMC10086258; doi:10.3389/fmicb.2023.1161155)
Supplement: Supplementary file 1 [file Table_1.DOCX]

Supplementary table A

| Application | Observations | References |
| --- | --- | --- |
| Reduction of upper respiratory tract bacterial and viral infections | Introducing strain BLIS K12, the prototype of the BLIS-producing oral probiotics | (Tagg and Dierksen, 2003) |
|  | Colonization of infants upper respiratory tract tissue with a pediatric formulation of BLIS K12 | (Power et al., 2008) |
|  | 41 children (3-12 y) completing a 90 day course of BLIS K12 showed 90% reduction in strep pharyngitis compared to equivalent time in previous year | (di Pierro et al., 2012) |
|  | 20 adults completing a 90 day course of BLIS K12 showed 80% reduction in strep pharyngitis compared to equivalent time in previous year | (di Pierro et al., 2013) |
|  | Children with recurrent pharyngotonsillitis treated (n=31) or untreated (n=30) with a 90 day course of BLIS K12. Significant infection reduction in treated compared to previous year | (di Pierro et al., 2014) |
|  | Children (3-4 y) either treated (n=111) or not treated (n=111) with daily BLIS K12 for 6 months. Strep pharyngitis, scarlet fever and otitis media present in 16%, 9% and 44% of the treated and in 48%, 4% and 80% of the controls. | (di Pierro et al., 2016b) |
|  | Children (3-7 y) having recurrent strep throat either given BLIS K12 for 90 days (n=76) or untreated (n=54). Treated children experienced significantly fewer strep infections both during the test period and in subsequent 9 month period than the control group. | (Gregori et al., 2016) |
|  | Children (3-10 y) either treated (n=48) or untreated (n=76) with BLIS K12 for 90 days. 90% reduction in strep pharyngitis in treated group compared to equivalent period of previous year. | (di Pierro et al., 2016c) |
|  | Teens and adults (12-45 y) experiencing recurrent strep pharyngitis either treated with BLIS K12 (n= 20) or control (n=14) for 3 months experienced 95% reduction in infections compared to previous year. | (Gun, 2017) |
|  | Children (3-14 y) (n=133) receiving BLIS K12 for 6 months experienced *ca.* 90% reduction in pharyngitis and *ca.* 70% reduction in AOM episodes. | (di Pierro et al., 2018) |
|  | Children (5-14 y) were given BLIS K12 (n=666) or placebo (n=648) for 1 y. A small (but non-significant) reduction in GAS positive throat swabs was detected in the probiotic group. Note: see Di Pierro (2019) for further comment. | (Doyle et al., 2018) |
|  | Children (3-14 y) given BLIS K12 experienced a significant decrease in episodes of strep and non-strep tonsillitis and also in AOM relapses. | (Kryuchko and Tkachenko, 2018) |
|  | Children (5-10 y) experiencing recurrent tonsillitis treated with (n=50) with BLIS K12 and untreated (n=50) for 3 months plus 12 month follow-up. Significant reduction in tonsillitis episodes and days absence form school in treated group. | (Marini et al., 2019) |
|  | Medical staff treated (n=98) or untreated (n=95) with BLIS K12 for 30 days. Respiratory tract infections were reduced by 64.8% in treated group compared to controls. | (Wang et al., 2021a) |
|  | Children (3-10 y) treated with BLIS K12 (n= 47 ) or untreated (n= 50) for 30 days. Incidence of URTI was significantly lower in the treated (14.9% ) than untreated (34.0% ) children during the study period. | (Guo et al., 2022) |
|  | URTI-prone children (12-15 y) either treated (n=20) or placebo-controlled (n=20) with a probiotic product (including BLIS K12 and BLIS M18) for 3 months showed significant reduction of oral and respiratory tract infections. | (Campanella et al., 2018) |
|  | Pharyngotonsillitis subjects treated 30 days either with standard prophylaxis (n=30) or with BLIS K12 supplementation (n=30) or BLIS K12 plus “tonsillar sanitation” (n=30) showed reduced morbidity with use of BLIS K12. | (Puhlik et al., 2021) |
| SARS-CoV-2 infection | Proposal to use BLIS K12 as an adjunct to help control viral lung infections and associated pneumonias and to improve host immune functions. | (di Pierro, 2020) |
|  | Preliminary observation of reduced detection rate of SARS-CoV-2 positivity in children taking BLIS K12. | (di Pierro and Colombo, 2021) |
|  | Beneficial clinical effects of use of BLIS K12 in hospitalized Covid -19 patients. | (di Pierro et al., 2022) |
| Otitis media | Otitis media susceptible children (n=19; age 6 m -5y) dosed with powdered formulation of BLIS K12 following amoxycillin pretreatment led to 33% being colonised. | (Power et al., 2008) |
|  | 41 children (3-12 y) completing a 90 day course of BLIS K12 showed 40% reduction in otitis media episodes compared to equivalent time in previous year. | (di Pierro et al., 2012) |
|  | Pilot feasibility study of oral application of BLIS K12 to effect a reduction of otitis media episodes in children. | (di Pierro et al., 2015) |
|  | Children (3-14 y) (n=133) receiving BLIS K12 for 6 months experienced ca. 90% reduction in pharyngitis and ca. 70% reduction in AOM episodes. | (di Pierro et al., 2018) |
|  | *In vitro* demonstration of killing of 48% of otitis media pathogens by BLIS K12. | (Chen et al., 2021) |
|  | A study of 48 pharyngotonsillitis-susceptible children showed taking of BLIS K12 twice daily for 30 days significantly reduced infection occurrence and school day loss. | (Kryuchko and Tkachenko, 2018) |
| Halitosis reduction | Demonstration of halitosis reduction associated with the use of BLIS K12 and *in vitro* activity of BLIS K12 against Gram positive halitosis-associated bacteria. | (Burton et al., 2006a) |
|  | Improved organoleptic test scores achieved in children using BLIS K12 following chlorhexidine mouthrinses. | (Masdea et al., 2012) |
|  | A study of children (n=208) with halitosis demonstrated efficacy of improvement in organoleptic scores by BLIS K12 augmented by mechanical and chemical oral hygiene practices. | (Jamali et al., 2016) |
|  | Prior removal of the tongue coating enhanced the efficacy of halitosis reduction by BLIS K12. | (He et al., 2020) |
|  | BLIS K12 (and also BLIS M18) decreased the levels of volatile sulphur compounds produced *in vitro* by *P. gingivalis* and *T. denticola*. | (Yoo et al., 2019) |
| Group B streptococcus | BLIS K12 reduced GBS vaginal colonization in mice and killed all tested GBS in *in vitro* assessments. | (Patras et al., 2015) |
| Periodontitis | BLIS K12 displayed strong *in vitro* inhibition of *P. gingivalis* . | (Jansen et al., 2021) |
|  | An invertebrate model system and human oral keratinocytes utilized to demonstrate protection afforded by BLIS K12 against proliferation of periodontal pathogens. | (Moman et al., 2020) |
|  | The consumption of BLIS K12 by mice decreased anaerobic bacterial accumulation in plaque and lessened periodontitis-induced alveolar bone resorption and attachment loss. | (Zhu et al., 2019) |
| Candida albicans infection | BLIS K12 interfered with *in vitro* attachment of candida and reduced infectivity in a mouse model. | (Ishijima et al., 2012) |
|  | Reduction of candida biofilm formation demonstrated by BLIS K12. | (James et al., 2016) |
|  | BLIS K12 enhanced mycological cure and shortened the treatment course when used together with conventional antifungal therapy. | (Hu et al., 2019) |
|  | BLIS K12 consumption for 30 d reduced denture stomatitis and candida counts in 25 adults (test) compared with 25 control subjects. | (Passariello et al., 2020) |
|  | BLIS K12 inhibited *C. albicans* aggregation, biofilm formation and dimorphism. | (Mokhtar et al., 2021) |
|  | BLIS K12 shown to be inhibitory to Candida biofilm formation. | (Rismayuddin et al., 2022) |
| Dental plaque modulation | A 4 week course of BLIS K12 resulted in a significant decrease in plaque accumulation for 15 test subjects cf. 16 taking placebo. | (Babina et al., 2022) |
|  | Cariogram evaluation demonstrated a significant reduction in caries risk in subjects taking a 3 month course of either BLIS K12 (n=11) or BLIS M18 (n=11) cf controls (n=12). | (Poorni et al., 2022) |
|  | A 30 d course of BLIS K12 and BLIS M18 reduced S. mutans counts and "improved” salivary pH levels. | (Srinivasan et al., 2022) |
| Beneficial modulation of the immune system | Elevated levels of interferon gamma in human saliva were detected following ingestion of BLIS K12. | (Laws et al., 2022) |
|  | Rapid interferon-gamma release from natural killer cells induced by BLIS K12. | (Bouwer et al., 2013) |
|  | When either BLIS K12 or BLIS M18were co-administered to gingival fibroblasts together with periodontal pathogens, they effected a significant reduction in both IL-6 and IL-8 release. | (MacDonald et al., 2021) |
|  | BLIS K12 downregulated the innate immune responses of human epithelial cells and thereby promotes host-microbe homeostasis. | (Cosseau et al., 2008) |
|  | Following ingestion of BLIS K12, changes were detected in the levels of IL8, IL10 and IL12 in blood samples taken at 1 and 7 days. | (Laws et al., 2021) |
|  | BLIS K12 significantly decreased the severity and incidence of disease in a mouse model of rheumatoid arthritis. | (Li et al., 2019) |
|  | BLIS K12 did not cause upregulation B7-H1 and B7-DC repectors on squamous cell carcinoma cells, whereas, *P. gingivalis* did. | (Groeger et al., 2011) |
| Oral biofilm composition | Two weeks dietary supplementation with a probiotic cocktail containing BLIS K12 significantly increased levels of salivary BLIS K12 but did not influence the overall oral microbiome composition. | (Cernioglo et al., 2021) |
|  | Cell-free supernatants of BLIS K12 inhibited growth of *S. mutans* and *Staph hominis* and biofilm formation by *Schaalia odontolytica* and *Enterobacter cloacae.* | (Stašková et al., 2021) |
|  | BLIS K12 treatment alleviated radiation-induced oral mucositis in mice and reconstituted the dysbiotic oral microbiota. | (Wang et al., 2021b) |
|  | BLIS K12 culture supernatants reduced the formation of *S. epidermidis* biofilms and also effected a reduction of preformed biofilms. | (Frickmann et al., 2018) |
|  | BLIS K12 countered biofilm formation by *S. intermedius* on a titanium model dental implant. | (Vacca et al., 2020) |
|  | When covered by *S. oralis* or BLIS K12 biofilms, epithelial cells in culture were protected from *S. pyogenes* adherence, internalization, and cytotoxic effects. | (Fiedler et al., 2013) |
|  | BLIS K12 reduced viability of *S. pyogenes* in pharyngeal biofilms without markedly disrupting the microcosm composition | (Humphreys and McBain, 2019) |
|  | In a randomized trial, a daily BLIS K12 dose for 1 month did not disturb the diversity of saliva or nasopharyngeal microbiomes in children attending daycare. A short-term increase in *S. salivarius* in the saliva microbiome and a decrease in Moraxella was detected. | (Sarlin et al., 2021) |
| Oral lichen planis | Four weeks daily dosing with BLIS K12 restored oral streptococcal levels and was similarly effective to use of corticosteroids for treatment of symptomatic oral lichen planis. | (Li et al., 2020) |
| PFAPA (Periodic Fever with Aphthous stomatitis, Pharyngitis, and Adenitis) syndrome | 90-day administration of strain K12 to 4 subjects reduced specific signs of PFAPA, leading also to a reduction in drug use. | (di Pierro et al., 2016a) |
| Psoriasis | Psoriasis patients (n=100) treated for 90 d. with BLIS K12 or untreated (n= 98). 83.7% of treated patients achieved a 100% improvement of their PASI score at 24 weeks. | (Zangrilli et al., 2022) |
| Functional foods | The anti-*S. pyogenes* activity of coconut milk was enhanced by 68% when fermented with BLIS K12 providing a new functional (anti-*S. pyogenes*) non-dairy product. | (Han et al., 2022) |
| Skin pathogens | Vacuum dried BLIS K12 enclosed between polymer membrane pads shown upon rehydration to effect inhibition of *Cutibacterium acnes*, *Staphylococcus aureus* and  *Pseudomonas aeruginosa*. | (Khalfallah et al., 2021) |
| Detection of colonization | In the individual studied, BLIS K12 could be detected on oral mucosal membranes for 3 weeks following initial colonisation using a RTPCR probe specific for salivaricin A. | (Horz et al., 2007) |
| Molecular profiling for functional foods | The authors characterized the global cellular constituents of BLIS K12 bacteria i.e. its proteins, lipids, polysaccharides, ribosomes, nucleic acids and cell wall constituents by differential scanning calorimetry and infrared spectroscopic techniques. Prebiotic tapioca starch modulated keystone metabolic events in BLIS K12 - increasing nucleic acid and polysaccharide synthesis and decreasing fatty acids. | (Gurbanov and Yildiz, 2017) |
|  | Tapioca starch modulated “keystone metabolic events” in a dose-dependent manner in batch cultures of BLIS K12. Expressions of these changes included increased cell counts and alterations to colony morphologies. Extensive molecular changes were also detected including protein metabolism and in protein secondary structures. | (Gurbanov et al., 2021) |
| Safety studies associated with oral ingestion | No adverse reactions detected. No overt change in microbiota composition or in blood clinical chemistry. | (Burton et al., 2006b, 2010, 2011) |
|  | Genome sequence of BLIS K12. | (Barretto et al., 2012) |
| Adenoiditis prophylaxis | Children with chronic adenoiditis were either treated (n=113) or control (n=106) with BLIS K12 for 30 d. decreased the frequency of exacerbations and reduced the need for medication therapy. | (Karpova et al., 2015) |
| Relative insensitivity of BLIS K12 to lentiscus oil | BLIS K12 (and also BLIS M18) appeared relatively less sensitive to lentiscus oil than strains of the pathogenic streptococcal species *S. agalactiae, S. intermedius, S. mitis, S. mutans, S. pyogenes.* | (Orrù et al., 2017) |
|  | Demonstrated increased activity against pathogenic streptococci (and not BLIS K12 or BLIS M18) using winterized lentiscus oil. | (di Pierro et al., 2021) |
| BLIS K12 growth in artificial saliva | Data derived to characterise the effects of exhaled air and three enzymes contained in saliva on the growth and physiological state of BLIS K12. | (Roger et al., 2011) |
| Other studies | Investigated the role of BLIS K12 in interfering with the adherence of pneumococci to human HEp-2 cells. | (Dunne et al., 2014) |
|  | Development of PCR detection methodology for BLIS K12 | (Reid et al., 2020) |
|  | BLIS K12 autoimmune modulation effective in a mouse model of rheumatoid arthritis. | (Li et al., 2022) |
|  | Speculation that suppression of NF-kB activity by BLIS K12 may have a role in the management of type 2 diabetes? | (Bhardwaj et al., 2020) |
|  | Demonstration that BLIS K12 and M18 can reduce pneumococcal adherence to pharyngeal epithelial cells using several mechanisms including blocking of binding sites. | (Manning et al., 2016) |
|  | Consumption of BLIS K12 did not reduce pain experienced post tonsillectomy and there was no significantly difference in the frequency of adverse events between those consuming BLIS K12 and placebo control. | (Nasserallah et al., 2022) |

References

Babina, K., Salikhova, D., Polyakova, M., Svitich, O., Samoylikov, R., Ahmad El-Abed, S., et al. (2022). The effect of oral probiotics (*Streptococcus salivarius* K12) on the salivary level of secretory immunoglobulin A, salivation rate, and oral biofilm: a pilot randomized clinical trial. *Nutrients* 14, 1124. doi: 10.3390/NU14051124.

Barretto, C., Alvarez-Martin, P., Foata, F., Renault, P., and Berger, B. (2012). Genome sequence of the lantibiotic bacteriocin producer *Streptococcus salivarius*  strain K12. *J Bacteriol* 194, 5959–5960. doi: 10.1128/JB.01268-12.

Bhardwaj, R., Singh, B. P., Sandhu, N., Singh, N., Kaur, R., Rokana, N., et al. (2020). Probiotic mediated NF-κB regulation for prospective management of type 2 diabetes. *Mol Biol Rep* 47, 2301–2313. doi: 10.1007/S11033-020-05254-4.

Bouwer, A. L., Saunderson, S. C., Dunn, A. C., Lester, K. L., Crowley, L. R., Jack, R. W., et al. (2013). Rapid interferon-gamma release from natural killer cells induced by a streptococcal commensal. *https://home.liebertpub.com/jir* 33, 459–466. doi: 10.1089/JIR.2012.0116.

Burton, J. P., Chilcott, C. N., Moore, C. J., Speiser, G., and Tagg, J. R. (2006a). A preliminary study of the effect of probiotic *Streptococcus salivarius* K12 on oral malodour parameters. *J Appl Microbiol* 100, 754–764. doi: 10.1111/j.1365-2672.2006.02837.x.

Burton, J. P., Chilcott, C. N., Wescombe, P. A., and Tagg, J. R. (2010). Extended safety data for the oral cavity probiotic S*treptococcus salivarius* K12. *Probiotics Antimicrob Proteins* 2, 135–144. doi: 10.1007/s12602-010-9045-4.

Burton, J. P., Cowley, S., Simon, R. R., McKinney, J., Wescombe, P. A., and Tagg, J. R. (2011). Evaluation of safety and human tolerance of the oral probiotic *Streptococcus salivarius* K12: a randomized, placebo-controlled, double-blind study. *Food Chem Toxicol* 49, 2356–2364. doi: 10.1016/j.fct.2011.06.038.

Burton, J. P., Wescombe, P. A., Moore, C. J., Chilcott, C. N., and Tagg, J. R. (2006b). Safety assessment of the oral cavity probiotic *Streptococcus salivarius* K12. *Appl Environ Microbiol* 72, 3050–3053. doi: 10.1128/AEM.72.4.3050-3053.2006.

Campanella, V., Syed, J., Santacroce, L., Saini, R., Ballini, A., and Inchingolo, F. (2018). Oral probiotics influence oral and respiratory tract infections in pediatric population: a randomized double-blinded placebo-controlled pilot study. *Eur Rev Med Pharmacol Sci* 22, 8034–8041. doi: 10.26355/EURREV_201811_16433.

Cernioglo, K., Kalanetra, K. M., Meier, A., Lewis, Z. T., Underwood, M. A., Mills, D. A., et al. (2021). Multi-strain probiotic supplementation with a product containing human-native *S. salivarius* K12 in healthy adults increases oral *S. salivarius*. *Nutrients* 13, 4392. doi: 10.3390/NU13124392.

Chen, T. Y., Hale, J. D. F., Tagg, J. R., Jain, R., Voss, A. L., Mills, N., et al. (2021). *In vitro* inhibition of clinical isolates of otitis media pathogens by the probiotic *Streptococcus salivarius* BLIS K12. *Probiotics Antimicrob Proteins* 13, 734–738. doi: 10.1007/S12602-020-09719-7.

Cosseau, C., Devine, D. A., Dullaghan, E., Gardy, J. L., Chikatamarla, A., Gellatly, S., et al. (2008). The commensal *Streptococcus salivarius* K12 downregulates the innate immune responses of human epithelial cells and promotes host-microbe homeostasis. *Infect Immun* 76, 4163–4175. doi: 10.1128/IAI.00188-08.

di Pierro, F. (2020). A possible probiotic (*S. salivarius* K12) approach to improve oral and lung microbiotas and raise defenses against SAR S-CoV-2. *Minerva Med* 111, 281–283. doi: 10.23736/S0026-4806.20.06570-2.

di Pierro, F., Adami, T., Rapacioli, G., Giardini, N., and Streitberger, C. (2013). Clinical evaluation of the oral probiotic *Streptococcus salivarius* K12 in the prevention of recurrent pharyngitis and/or tonsillitis caused by *Streptococcus pyogenes* in adults. *Expert Opin Biol Ther* 13, 339–343. doi: 10.1517/14712598.2013.758711.

di Pierro, F., Campana, A., and Panatta, M. L. (2016a). The use of *Streptococcus salivarius* K12 in attenuating PFAPA syndrome, a pilot study. *Altern Integr Med* 05. doi: 10.4172/2327-5162.1000222.

di Pierro, F., and Colombo, M. (2021). The administration of S*. salivarius* K12 to children may reduce the rate of SARS-CoV-2 infection. *Minerva Med* 112. doi: 10.23736/S0026-4806.21.07487-5.

di Pierro, F., Colombo, M., Giuliani, M. G., Danza, M. L., Basile, I., Bollani, T., et al. (2016b). Effect of administration of S*treptococcus salivariu*s K12 on the occurrence of streptococcal pharyngo-tonsillitis, scarlet fever and acute otitis media in 3 years old children. *Eur Rev Med Pharmacol Sci* 20, 4601–4606.

di Pierro, F., Colombo, M., Zanvit, A., Risso, P., and Rottoli, A. S. (2014). Use of *Streptococcus salivarius* K12 in the prevention of streptococcal and viral pharyngotonsillitis in children. *Drug Healthc Patient Saf* 6, 15–20. doi: 10.2147/dhps.s59665.

di Pierro, F., Colombo, M., Zanvit, A., and Rottoli, A. S. (2016c). Positive clinical outcomes derived from using *Streptococcus salivarius* K12 to prevent streptococcal pharyngotonsillitis in children: a pilot investigation. *Drug Healthc Patient Saf* 8, 77–81. doi: 10.2147/DHPS.S117214.

di Pierro, F., di Pasquale, D., and di Cicco, M. (2015). Oral use of *Streptococcus salivarius* K12 in children with secretory otitis media: preliminary results of a pilot, uncontrolled study. *Int J Gen Med* 8, 303–308. doi: 10.2147/IJGM.S92488.

di Pierro, F., Donato, G., Fomia, F., Adami, T., Careddu, D., Cassandro, C., et al. (2012). Preliminary pediatric clinical evaluation of the oral probiotic *Streptococcus salivariu*s K12 in preventing recurrent pharyngitis and/or tonsillitis caused by *Streptococcus pyogenes* and recurrent acute otitis media. *Int J Gen Med* 5, 991–997. doi: 10.2147/IJGM.S38859.

di Pierro, F., Iqtadar, S., Mumtaz, S. U., Bertuccioli, A., Recchia, M., Zerbinati, N., et al. (2022). Clinical effects of *Streptococcus salivarius* K12 in hospitalized COVID-19 patients: results of a preliminary study. *Microorganisms* 10, 1926. doi: 10.3390/microorganisms10101926.

di Pierro, F., Risso, P., Poggi, E., Timitilli, A., Bolloli, S., Bruno, M., et al. (2018). Use of S*treptococcus salivarius* K12 to reduce the incidence of pharyngo-tonsillitis and acute otitis media in children: a retrospective analysis in not-recurrent pediatric subjects. *Minerva Pediatr* 70, 240–245. doi: 10.23736/S0026-4946.18.05182-4.

di Pierro, F., Sagheddu, V., Galletti, S., Forti, M., Elli, M., Bertuccioli, A., et al. (2021). Antibacterial activity of a fractionated Pistacia lentiscus oil against pharyngeal and ear pathogens, alone or in combination with antibiotics. *Front Microbiol* 12, 1587. doi: 10.3389/FMICB.2021.686942/BIBTEX.

Doyle, H., Pierse, N., Tiatia, R., Williamson, D., Baker, M., and Crane, J. (2018). Effect of oral probiotic *Streptococcus salivarius* K12 on group A streptococcus  pharyngitis: a pragmatic trial in schools. *Pediatr Infect Dis J* 37, 619–623. doi: 10.1097/INF.0000000000001847.

Dunne, E. M., Toh, Z. Q., John, M., Manning, J., Satzke, C., and Licciardi, P. (2014). Investigating the effects of probiotics on pneumococcal colonization using an in vitro adherence assay. *J Vis Exp*. doi: 10.3791/51069.

Fiedler, T., Riani, C., Koczan, D., Standar, K., Kreikemeyer, B., and Podbielski, A. (2013). Protective mechanisms of respiratory tract streptococci against *Streptococcus pyogenes* biofilm formation and epithelial cell infection. *Appl Environ Microbiol* 79, 1265. doi: 10.1128/AEM.03350-12.

Frickmann, H., Klenk, C., Warnke, P., Redanz, S., and Podbielski, A. (2018). Influence of probiotic culture supernatants on *in vitro* biofilm formation of staphylococci. *Eur J Microbiol Immunol (Bp)* 8, 119. doi: 10.1556/1886.2018.00022.

Gregori, G., Righi, O., Risso, P., Boiardi, G., Demuru, G., Ferzetti, A., et al. (2016). Reduction of group A beta-hemolytic streptococcus pharyngo-tonsillar infections associated with use of the oral probiotic *Streptococcus salivarius* K12: a retrospective observational study. *Ther Clin Risk Manag* 12, 87. doi: 10.2147/TCRM.S96134.

Groeger, S., Domann, E., Gonzales, J. R., Chakraborty, T., and Meyle, J. (2011). B7-H1 and B7-DC receptors of oral squamous carcinoma cells are upregulated by *Porphyromonas gingivalis*. *Immunobiology* 216, 1302–1310. doi: 10.1016/J.IMBIO.2011.05.005.

Gun, T. (2017). Clinical evaluation of the therapeutic use of oral probiotic *Streptococcus salivarius* K12 for recurrent pharyngitis and/or tonsillitis. *Paripex - Indian Journal of Research* 6, 633–644.

Guo, H., Xiang, X., Lin, X., Wang, Q., Qin, S., Lu, X., et al. (2022). Oropharyngeal probiotic ENT-K12 as an effective dietary intervention for children with recurrent respiratory tract infections during cold season. *Front Nutr* 9. doi: 10.3389/fnut.2022.900448.

Gurbanov, R., Karadağ, H., Karaçam, S., and Samgane, G. (2021). Tapioca starch modulates cellular events in oral probiotic *Streptococcus salivarius* strains. *Probiotics Antimicrob Proteins* 13, 195–207. doi: 10.1007/S12602-020-09678-Z.

Gurbanov, R., and Yildiz, F. (2017). Molecular profile of oral probiotic bacteria to be used with functional foods. *J Food Health Sci* 3, 117–131. doi: 10.3153/JFHS17015.

Han, C. E., Ewe, J. A., Kuan, C. S., and Yeo, S. K. (2022). Growth characteristic of probiotic in fermented coconut milk and the antibacterial properties against *Streptococcus pyogenes*. *J Food Sci Technol* 59, 3379–3386. doi: 10.1007/S13197-021-05321-Z.

He, L., Yang, H., Chen, Z., and Ouyang, X. (2020). The effect of *Streptococcus salivarius* K12 on halitosis: a double-blind, randomized, placebo-controlled trial. *Probiotics Antimicrob Proteins* 12, 1321–1329. doi: 10.1007/S12602-020-09646-7.

Horz, H. P., Meinelt, A., Houben, B., and Conrads, G. (2007). Distribution and persistence of probiotic *Streptococcus salivarius* K12 in the human oral cavity as determined by real-time quantitative polymerase chain reaction. *Oral Microbiol Immunol* 22, 126–130. doi: 10.1111/j.1399-302X.2007.00334.x.

Hu, L., Mao, Q., Zhou, P., Lv, X., Hua, H., and Yan, Z. (2019). Effects of *Streptococcus salivarius* K12 with nystatin on oral candidiasis—RCT. *Oral Dis* 25, 1573–1580. doi: 10.1111/odi.13142.

Humphreys, G. J., and McBain, A. J. (2019). Antagonistic effects of *Streptococcus* and *Lactobacillus* probiotics in pharyngeal biofilms. *Lett Appl Microbiol* 68, 303–312. doi: 10.1111/LAM.13133.

Ishijima, S. A., Hayama, K., Burton, J. P., Reid, G., Okada, M., Matsushita, Y., et al. (2012). Effect of *Streptococcus salivarius* K12 on the i*n vitro* growth of *Candida albicans* and its protective effect in an oral candidiasis model. *Appl Environ Microbiol* 78, 2190–2199. doi: 10.1128/AEM.07055-11.

Jamali, Z., Aminabadi, N. A., Samiei, M., Deljavan, A. S., Shokravi, M., and Shirazi, S. (2016). Impact of chlorhexidine pretreatment followed by probiotic S*treptococcus salivarius* strain K12 on halitosis in children: A randomised controlled clinical trial. *Oral Health Prev Dent* 14, 305–313. doi: 10.3290/j.ohpd.a36521.

James, K. M., MacDonald, K. W., Chanyi, R. M., Cadieux, P. A., and Burton, J. P. (2016). Inhibition of *Candida albicans* biofilm formation and modulation of gene expression by probiotic cells and supernatant. *J Med Microbiol* 65, 328–336. doi: 10.1099/JMM.0.000226.

Jansen, P. M., Abdelbary, M. M. H., and Conrads, G. (2021). A concerted probiotic activity to inhibit periodontitis-associated bacteria. *PLoS One* 16. doi: 10.1371/JOURNAL.PONE.0248308.

Karpova, E. P., Karpycheva, I. E., and Tulupov, D. A. (2015). [Prophylaxis of chronic adenoiditis in the children]. *Vestn Otorinolaringol* 80, 43–45. doi: 10.17116/otorino201580643-45.

Khalfallah, G., Gartzen, R., Möller, M., Heine, E., and Lütticken, R. (2021). A new approach to harness probiotics against common bacterial skin pathogens: towards living antimicrobials. *Probiotics Antimicrob Proteins* 1, 3. doi: 10.1007/s12602-021-09783-7.

Kryuchko, T. O., and Tkachenko, O. Ya. (2018). Clinical experience of *Streptococcus salivarius* K12 use for the prevention of pharyngotonsillitis and respiratory infections in children. *Child’s Health* 13, 629–634. doi: 10.22141/2224-0551.13.7.2018.148915.

Laws, G. A., Harold, L. K., Tagg, J. R., and Hale, J. D. F. (2022). Interferon gamma response in human saliva following exposure to the oral probiotic *Streptococcus salivarius* BLIS K12. *Probiotics Antimicrob Proteins*. doi: 10.1007/S12602-022-10010-0.

Laws, G. L., Hale, J. D. F., and Kemp, R. A. (2021). Human systemic immune response to ingestion of the oral probiotic *Streptococcus salivarius* BLIS K12. *Probiotics Antimicrob Proteins*. doi: 10.1007/s12602-021-09822-3.

Li, J., Jin, J., Li, S., Zhong, Y., Jin, Y., Zhang, X., et al. (2022). Tonsillar microbiome-derived lantibiotics induce structural changes of IL-6 and IL-21 receptors and modulate host immunity. *Advanced Science* 9, 2202706. doi: 10.1002/ADVS.202202706.

Li, J., Li, S., Jin, J., Guo, R., Sun, X., Guo, J., et al. (2019). Mining the human tonsillar microbiota as autoimmune modulator. *bioRxiv*, 719807. doi: 10.1101/719807.

Li, Y., Shao, F., Zheng, S., Tan, Z., and He, Y. (2020). Alteration of *Streptococcus salivarius* in buccal mucosa of oral lichen planus and controlled clinical trial in OLP treatment. *Probiotics Antimicrob Proteins* 12, 1340–1348. doi: 10.1007/S12602-020-09664-5.

MacDonald, K., Chanyi, R., Macklaim, J., Cadieux, P., Reid, G., and Burton, J. (2021). *Streptococcus salivarius* inhibits immune activation by periodontal disease pathogens. *BMC Oral Health* 21. doi: 10.1186/S12903-021-01606-Z.

Manning, J., Dunne, E. M., Wescombe, P. A., Hale, J. D. F., Mulholland, E. K., Tagg, J. R., et al. (2016). Investigation of *Streptococcus salivarius*-mediated inhibition of pneumococcal adherence to pharyngeal epithelial cells. *BMC Microbiol* 16, 1–9. doi: 10.1186/S12866-016-0843-Z/FIGURES/5.

Marini, G., Sitzia, E., Panatta, M. L., and de Vincentiis, G. C. (2019). Pilot study to explore the prophylactic efficacy of oral probiotic *Streptococcus  salivarius* K12 in preventing recurrent pharyngo-tonsillar episodes in pediatric patients. *Int J Gen Med* 12, 213–217. doi: 10.2147/IJGM.S168209.

Masdea, L., Kulik, E. M., Hauser-Gerspach, I., Ramseier, A. M., Filippi, A., and Waltimo, T. (2012). Antimicrobial activity of *Streptococcus salivarius* K12 on bacteria involved in oral malodour. *Arch Oral Biol* 57, 1041–1047. doi: 10.1016/j.archoralbio.2012.02.011.

Mokhtar, M., Rismayuddin, N. A. R., Mat Yassim, A. S., Ahmad, H., Abdul Wahab, R., Dashper, S., et al. (2021). *Streptococcus salivarius* K12 inhibits *Candida albicans* aggregation, biofilm formation and dimorphism. *Biofouling* 37, 767–776. doi: 10.1080/08927014.2021.1967334.

Moman, R., O’Neill, C. A., Ledder, R. G., Cheesapcharoen, T., and McBain, A. J. (2020). Mitigation of the toxic effects of periodontal pathogens by candidate probiotics in oral keratinocytes, and in an invertebrate model. *Front Microbiol* 11. doi: 10.3389/FMICB.2020.00999.

Nasserallah, M. v, de Silva, N. M., Tobin, V., Rozen, W. M., and Hunter-Smith, D. J. (2022). Can probiotic gargles reduce post-tonsillectomy morbidity in adult patients? A pilot, triple-blinded, randomised controlled trial and feasibility study. *J Laryngol Otol*, 1–41. doi: 10.1017/s0022215122000743.

Orrù, G., Demontis, C., Mameli, A., Tuveri, E., Coni, P., Pichiri, G., et al. (2017). The selective interaction of *Pistacia lentiscus* oil vs. human streptococci, an old functional food revisited with new tools. *Front Microbiol* 8. doi: 10.3389/FMICB.2017.02067.

Passariello, C., di Nardo, F., Polimeni, A., di Nardo, D., and Testarelli, L. (2020). Probiotic *Streptococcus salivarius* reduces symptoms of denture stomatitis and oral colonization by *Candida albicans*. *Applied Sciences* 10, 3002. doi: 10.3390/app10093002.

Patras, K. A., Wescombe, P. A., Rösler, B., Hale, J. D., Tagg, J. R., and Doran, K. S. (2015). *Streptococcus salivarius* K12 limits group B streptococcus vaginal colonization. *Infect Immun* 83, 3438–3444. doi: 10.1128/IAI.00409-15.

Poorni, S., Nivedhitha, M., Srinivasan, M., and Balasubramaniam, A. (2022). Effect of probiotic *Streptococcus salivarius* K12 and M18 lozenges on the cariogram parameters of patients with high caries risk: a randomised control trial. *Cureus*. doi: 10.7759/cureus.23282.

Power, D., Burton, J., Chilcott, C., Dawes, P., and Tagg, J. (2008). Preliminary investigations of the colonisation of upper respiratory tract tissues of infants using a paediatric formulation of the oral probiotic *Streptococcus salivarius* K12. *Eur J Clin Microbiol Infect Dis* 27, 1261–1263. doi: 10.1007/S10096-008-0569-4.

Puhlik, S. M., Аndreev, ;, А V, Gushcha, ;, Tagunova, ;, I К, et al. (2021). Experience with the use of oral probiotic *Streptococcus salivarius* K12 for the prevention of recurrence of pharyngotonsillar episodes. 1, 120–124. Available at: http://pharmacologyonline.silae.it.

Reid, P., Heng, N. C. K., Hale, J. D., Krishnan, D., Crane, J., Tagg, J. R., et al. (2020). A TaqMan^TM^-based quantitative PCR screening assay for the probiotic *Streptococcus salivarius* K12 based on the specific detection of its megaplasmid-associated salivaricin B locus. *J Microbiol Methods* 170. doi: 10.1016/j.mimet.2020.105837.

Rismayuddin, N. A. R., Mohd Badri, P. E. A., Ismail, A. F., Othman, N., Bandara, H. M. H. N., and Arzmi, M. H. (2022). Synbiotic *Musa acuminata* skin extract and *Streptococcus salivarius* K12 inhibit candida species biofilm formation. *Biofouling*, 1–14. doi: 10.1080/08927014.2022.2105142.

Roger, P., Harn-Arsa, S., Delettre, J., and Béal, C. (2011). Salivary enzymes and exhaled air affect *Streptococcus salivarius* growth and physiological state in complemented artificial saliva. *Arch Microbiol* 193, 905–910. doi: 10.1007/S00203-011-0746-1.

Sarlin, S., Tejesvi, M. v., Turunen, J., Vänni, P., Pokka, T., Renko, M., et al. (2021). Impact of *Streptococcus salivarius* K12 on nasopharyngeal and saliva microbiome: a randomized controlled trial. *Pediatric Infectious Disease Journal*, 394–402. doi: 10.1097/INF.0000000000003016.

Stašková, A., Sondorová, M., Nemcová, R., Kačírová, J., Mad’ar, M., and Karygianni, L. (2021). Antimicrobial and antibiofilm activity of the probiotic strain *Streptococcus salivarius* K12 against oral potential pathogens. doi: 10.3390/antibiotics10070793.

Tagg, J., and Dierksen, K. (2003). Bacterial replacement therapy: adapting “germ warfare” to infection prevention. *Trends Biotechnol* 21, 217–223. doi: 10.1016/S0167-7799(03)00085-4.

Vacca, C., Contu, M. P., Rossi, C., Ferrando, M. L., Blus, C., Szmukler-Moncler, S., et al. (2020). *In vitro* Interactions between *Streptococcus intermedius* and *Streptococcus salivarius* K12 on a titanium cylindrical surface. *Pathogens* 9, 1–15. doi: 10.3390/PATHOGENS9121069.

Wang, Q., Lin, X., Xiang, X., Liu, W., Fang, Y., Chen, H., et al. (2021a). Oropharyngeal probiotic ENT-K12 prevents respiratory tract infections among frontline medical staff fighting against COVID-19: A Pilot Study. *Front Bioeng Biotechnol* 9. doi: 10.3389/fbioe.2021.646184.

Wang, Y., Li, J., Zhang, H., Zheng, X., Wang, J., Jia, X., et al. (2021b). Probiotic *Streptococcus salivarius* K12 alleviates radiation-induced oral mucositis in mice. *Front Immunol* 12. doi: 10.3389/fimmu.2021.684824.

Yoo, J. il, Shin, I. S., Jeon, J. G., Yang, Y. M., Kim, J. G., and Lee, D. W. (2019). The effect of probiotics on halitosis: a systematic review and meta-analysis. *Probiotics Antimicrob Proteins* 11, 150–157. doi: 10.1007/s12602-017-9351-1.

Zangrilli, A., Diluvio, L., di Stadio, A., and di Girolamo, S. (2022). Improvement of psoriasis using oralpProbiotic *Streptococcus salivarius* K-12: a case-control 24-month longitudinal study. *Probiotics Antimicrob Proteins* 14, 573–578. doi: 10.1007/S12602-022-09937-1.

Zhu, L., Li, H., Yang, X., Xue, L., Li, X., and Du, J. (2019). Effects of *Streptococcus salivarius* K12 on experimental periodontitis and oral microbiota in mice. *J Biosci Med (Irvine)* 07, 95–111. doi: 10.4236/jbm.2019.712009.
