# Supplementary material for: Beneficial modulation of human health in the oral cavity and beyond using bacteriocin-like inhibitory substance-producing streptococcal probiotics
Source: Front Microbiol. 2023 Mar 28;14:1161155. doi: 10.3389/fmicb.2023.1161155 (PMC10086258; doi:10.3389/fmicb.2023.1161155)
Supplement: Supplementary file 2 [file Table_2.DOCX]

Supplementary table B

| Application | Observation | References |
| --- | --- | --- |
| Cancer cells | Inulin-supplemented BLIS M18 culture supernatants displayed anti-cancer cell activity in vitro. | (Karaçam and Tunçer, 2022) |
| Influence on indices of dental health | Randomized, double blind placebo controlled 3-month trial in 100 caries active children. Lowered plaque scores and no adverse events. Mutans strep counts were lowered in subjects well colonized with BLIS M18 | (Burton et al., 2013b) |
| Cariogram impact | High caries risk children (age 6-17 y) randomised to take BLIS M18 (n= 38) or control (n=38) for 90 d. Cariogram analysis indicated taking BLIS M18 increases the chances of avoiding new dental caries development | (di Pierro et al., 2015) |
| Gingivitis/periodontitis | Subjects with moderate gingivitis and periodontitis using BLIS M18 lozenges (n=14) for 30 d showed significant improvement cf control subjects (n=14) in all periodontal paraments | (Scariya et al., 2015) |
| Black stains on teeth | BLIS M18 culture supernatant interfered with the *in vitro* growth of two plaque species associated with black stain (bs) formation | (Gobbi et al., 2020) |
| Black stains on teeth  Oral cavity colonization | Children with a history of forming bs plaque were treated (n=28) with BLIS M18 for 3 months or controls (n=26 ). A significant difference (p < 0.05) in numbers of children with bs plaque at 3 m. | (Bardellini et al., 2020) |
|  | 75 subjects received four different doses of BLIS M18 for 28 days. Probiotic persistence was dependent upon the dose, but not the period of administration.  The overall composition of the oral microbiome was not modified by the probiotic treatment. | (Burton et al., 2013a) |
| Ps aeruginosa and K. pneumoniae growth inhibition | BLIS M18 culture supernatants reduced the growth *in vitro* of Ps. aeruginosa and Klebsiella pneumoniae | (Tunçer and Karaçam, 2020) |
| Post radiotherapy application | Randomised, double-blind, placebo-controlled 4 wk trial of BLIS M18 in 7 (test) and 6 (placebo) head and neck cancer patients did not detect substantial change in the salivary or plaque abundance of S. salivarius or mutans streptococci. | (Vesty et al., 2020) |
| Periodontal pathogens in vitro | BLIS M18 exhibited strong *in vitro* inhibitory activity against a series of periodontal pathogens. P. gingivalis and F. nucleatum were best inhibited by pure cultures of BLISK12 or BLISM18, respectively. | (Jansen et al., 2021) |
| S. mutans levels | Study compared probiotic milk and probiotic lozenges (included BLIS M18) | (Sengupta et al., 2020) |
| Cariogram levels | A statistically significant reduction in salivary *S. mutans* levels (p=0.001) and an increase in the salivary pH (p = 0.001) was observed after the use of either BLIS M18 (n= 22)or BLIS K12 (n= 21) for 30 d. when compared to the baseline. Controls (n=20) showed no significant change. | (Srinivasan et al., 2022) |
| Cariogram levels  Lentiscus oil insensitivity | Cariogram analysis of subjects using BLIS M18 (n=) , BLIS K12 (n=) or control (n=) for 3 m. demonstrated a considerable reduction in caries risk, especially for those taking BLIS K12 | (Poorni et al., 2022) |
|  | 60 day controlled trial reduced plaque accumulation and calculus formation in high risk caries population | (Tandelilin et al., 2018) |
|  | BLIS K12 and BLIS M18 were less sensitive to lentiscus oil than strains of five different pathogenic species *S. agalactiae, S. intermedius, S. mitis, S. mutans, S. pyogenes*. | (Orrù et al., 2017) |
| Tapioca starch fermentation | Tapioca starch modulated “keystone metabolic events” in a dose-dependent manner in batch cultures of BLIS M18. Expressions of these changes included increased cell counts and alterations to colony morphologies. Extensive molecular changes were also detected including within protein metabolism and protein secondary structures. Nucleic acid and polysaccharide synthesis increased and fatty acids were diminished. | (Gurbanov et al., 2021) |
| In vitro activity against S. mutans | BLIS M18 was shown to have relatively stronger inhibitory activity *in vitro* against *S. mutans* than BLIS K12 | (Chandrasekhar et al., 2020) |
| Halitosis | BLIS K12 and BLIS M18 were both effective at decreasing the levels of volatile sulphur compounds produced in vitro by *P. gingivalis* and *T. denticola*. | (Yoo et al., 2020) |
| Halitosis  Safety assessment | Triple blind placebo controlled trial in patients undergoing fixed orthodontic braces. Probiotic (n = 32), Placebo (n =32) group taking twice daily lozenges for 1 month with 3 month follow up. *S. salivarius* M18 reduced the level of halitosis in patients with orthodontic braces | (Benic et al., 2019) |
|  | Direct comparison of the BLIS M18 safety profile with that of BLIS K12 supports its safe application as a probiotic in humans. | (Hale et al., 2022) |
| Safety assessment | Genome sequence | (Heng et al., 2011) |
|  |  |  |

References

Bardellini, E., Amadori, F., Gobbi, E., Ferri, A., Conti, G., and Majorana, A. (2020). Does *Streptococcus salivarius* strain M18 assumption make black stains disappear in children? *Oral Health Prev Dent* 18, 161–164. doi: 10.3290/j.ohpd.a43359.

Benic, G. Z., Farella, M., Morgan, X. C., Viswam, J., Heng, N. C., Cannon, R. D., et al. (2019). Oral probiotics reduce halitosis in patients wearing orthodontic braces: a randomized, triple-blind, placebo-controlled trial. *J Breath Res* 13, 36010. doi: 10.1088/1752-7163/AB1C81.

Burton, J. P., Drummond, B. K., Chilcott, C. N., Tagg, J. R., Thomson, W. M., Hale, J. D. F., et al. (2013a). Influence of the probiotic *Streptococcus salivarius* strain M18 on indices of dental health in children: A randomized double-blind, placebo-controlled trial. *J Med Microbiol* 62, 875–884. doi: 10.1099/jmm.0.056663-0.

Burton, J. P., Wescombe, P. A., Macklaim, J. M., Chai, M. H. C., MacDonald, K., Hale, J. D. F., et al. (2013b). Persistence of the oral probiotic *Streptococcus salivarius* M18 is dose dependent and megaplasmid transfer can augment their bacteriocin production and adhesion Characteristics. *PLoS One* 8. doi: 10.1371/JOURNAL.PONE.0065991.

Chandrasekhar, S. N., Mallikarjun, S. B., and Salim, H. P. (2020). Comparative Evaluation of antibacterial activity of probiotics SK12 and SM18: An i*n vitro* Study. *Int J Clin Pediatr Dent* 13, 611–616. doi: 10.5005/jp-journals-10005-1838.

di Pierro, F., Zanvit, A., Nobili, P., Risso, P., and Fornaini, C. (2015). Cariogram outcome after 90 days of oral treatment with *Streptococcus salivarius* M18 in children at high risk for dental caries: results of a randomized, controlled study. *Clin Cosmet Investig Dent* 7, 107–113. doi: 10.2147/CCIDE.S93066.

Gobbi, E., de Francesco, M. A., Piccinelli, G., Caruso, A., Bardellini, E., and Majorana, A. (2020). In vitro inhibitory effect of two commercial probiotics on chromogenic actinomycetes. *Eur Arch Paediatr Dent* 21, 673–677. doi: 10.1007/S40368-020-00512-2.

Gurbanov, R., Karadağ, H., Karaçam, S., and Samgane, G. (2021). Tapioca starch modulates cellular events in oral probiotic *Streptococcus salivarius* strains. *Probiotics Antimicrob Proteins* 13, 195–207. doi: 10.1007/S12602-020-09678-Z.

Hale, J. D. F., Jain, R., Wescombe, P. A., Burton, J. P., Simon, R. R., and Tagg, J. R. (2022). Safety assessment of *Streptococcus salivarius* M18 a probiotic for oral health. *Benef Microbes*, 1–14. doi: 10.3920/BM2021.0107.

Heng, N. C. K., Haji-Ishak, N. S., Kalyan, A., Wong, A. Y. C., Lovrić, M., Bridson, J. M., et al. (2011). Genome sequence of the bacteriocin-producing oral probiotic *Streptococcus salivarius* strain M18. *J Bacteriol*. doi: 10.1128/JB.06001-11.

Jansen, P. M., Abdelbary, M. M. H., and Conrads, G. (2021). A concerted probiotic activity to inhibit periodontitis-associated bacteria. *PLoS One* 16. doi: 10.1371/JOURNAL.PONE.0248308.

Karaçam, S., and Tunçer, S. (2022). Exploiting the acidic extracellular pH: evaluation of *Streptococcus salivarius* M18 postbiotics to target cancer cells. *Probiotics Antimicrob Proteins* 14. doi: 10.1007/S12602-021-09806-3.

Orrù, G., Demontis, C., Mameli, A., Tuveri, E., Coni, P., Pichiri, G., et al. (2017). The selective interaction of *Pistacia lentiscus* oil vs. human streptococci, an old functional food revisited with new tools. *Front Microbiol* 8. doi: 10.3389/FMICB.2017.02067.

Poorni, S., Nivedhitha, M., Srinivasan, M., and Balasubramaniam, A. (2022). Effect of probiotic *Streptococcus salivarius* K12 and M18 lozenges on the cariogram parameters of patients with high caries risk: a randomised control trial. *Cureus*. doi: 10.7759/cureus.23282.

Scariya, L., Nagarathna, D. v, and Varghese, M. (2015). Probiotics in periodontal therapy. *Int J Pharma Bio Sci* 6, P242--P250. doi: 10.5455/musbed.20141106034910.

Sengupta, P., Desai, P. D., Maity, I., Mazumdar, P., Biswas, S., Choudhury, S. R., et al. (2020). Comparative evaluation of different probiotic products on salivary *Streptococcus mutans* and *Lactobacillus* level in caries risk population. *J Conserv Dent* 23, 619–623. doi: 10.4103/JCD.JCD_467_20.

Srinivasan, M., MS, N., and Poorni, S. (2022). Comparing the effect of probiotic *Streptococcus salivarius* K12 and M18 on the *Streptococcus mutans* count, salivary pH and buffer capacity: a randomized double blinded clinical trial. *Cumhuriyet Dental Journal*, 346–354. doi: 10.7126/cumudj.941928.

Tandelilin, ReginaT. C., Widita, E., Agustina, D., and Saini, R. (2018). The effect of oral probiotic consumption on the caries risk factors among high-risk caries population. *Journal of International Oral Health* 10, 132. doi: 10.4103/jioh.jioh_82_18.

Tunçer, S., and Karaçam, S. (2020). Cell-free supernatant of *Streptococcus salivarius* M18 impairs the pathogenic properties of *Pseudomonas aeruginosa* and *Klebsiella pneumonia*. *Arch Microbiol* 202, 2825–2840. doi: 10.1007/S00203-020-02005-8.

Vesty, A., Gear, K., Boutell, S., Taylor, M. W., Douglas, R. G., and Biswas, K. (2020). Randomised, double-blind, placebo-controlled trial of oral probiotic *Streptococcus salivarius* M18 on head and neck cancer patients post-radiotherapy: a pilot study. *Sci Rep* 10. doi: 10.1038/S41598-020-70024-Y.

Yoo, H. J., Jwa, S. K., Kim, D. H., and Ji, Y. J. (2020). Inhibitory effect of *Streptococcus salivarius* K12 and M18 on halitosis i*n vitro*. *Clin Exp Dent Res* 6, 207–214. doi: 10.1002/cre2.269.
