# Supplementary material for: Beneficial modulation of human health in the oral cavity and beyond using bacteriocin-like inhibitory substance-producing streptococcal probiotics
Source: Front Microbiol. 2023 Mar 28;14:1161155. doi: 10.3389/fmicb.2023.1161155 (PMC10086258; doi:10.3389/fmicb.2023.1161155)
Supplement: Supplementary file 3 [file Table_3.DOCX]

Supplementary table C

| *Streptococcus* *salivarius* strain(s) | Observations/Comments | References |
| --- | --- | --- |
| AMBR024, AMBR037, AMBR047, AMBR055, AMBR074, AMBR075 and AMBR158 | *In vitro* antagonistic activity demonstrated against classic bacterial otopathogens | (Jörissen et al., 2021) |
| ST48HK, ST59HK, ST61HK and ST62HK | Antagonistic properties, safety assessments and survival in the presence of commonly used drugs and oral hygiene products | (Kim et al., 2022) |
| LAB813 | Genome sequence included megaplasmid | (Gong et al., 2019) |
| 5M6c | Producer of salivaricin D | (Birri et al., 2012) |
| BP8, BP156 and BP160 | Preliminary assessments of role in functional foods and potential for cancer treatment | (Srikham et al., 2021) |
| HS0302 | Draft genome sequence – megaplasmid encodes two lantibiotics | (Geng et al., 2019) |
| JF | Draft genome sequence - genes encoding bacteriocins BlpU and colicin V | (Jia, 2016) |
| DPC6487 | Gut isolate shown to produce nisin G | (Lawrence et al., 2022a) |
| DPC6993 | Gut isolate producing salivaricins A5 and B. Targets *F. nucleatum* in colon fermentation model | (Lawrence et al., 2022b) |
| 24SMB | Selected as probiotic candidate on basis of safety profile, adhesion to Hep-2 cells and inhibition of *S. pneumoniae* | (Santagati et al., 2012) |
|  | Nasal bacteriotherapy with strain 24SMB used to treat urti and sleep disordered breathing in children. | (Bellussi et al., 2018) |
|  | Nasal spray bacteriotherapy together with *S. oralis* 89a (a) reduced and (b) treated respiratory infections and (c) reduced the requirement for adenoidectomy in children. | a.(Tarantino et al., 2018)  b. (Manti et al., 2020)  c. (Mantia et al., 2019) |
|  | Beneficial modulation of nasal microbiota was demonstrated associated with use of the probiotic nasal spray | (Grandi et al., 2019) |
|  | Genome sequence published | (Vertillo Aluisio et al., 2022) |
| K28 | Producer of enocin, a competitive inhibitor of pantothenate utilization by *S. pyogenes* | (Sanders and Sanders, 1982) |
| MS-oral-D6 | Promoted gingival re-epithelialization *in vitro* | (Fernandez-Gutierrez et al., 2017) |
| DB-B5 | Genome announcement | (Fields et al., 2020) |
|  | Safety assessment | (Li et al., 2021) |
| NBRC13956 | Piglet gut isolate. Safety /adhesion characteristics studied | (Dlamini et al., 2019) |
| JH | Producer of multiple bacteriocins and strong dextranase activity | (Walker et al., 2016) |
| ST3 and RS1 | Bound *in vitro* to human pharyngeal cells and modulated NF-B activation | (Guglielmetti et al., 2010) |
|  | Suggested probiotic application of ST3 together with *L. helveticus* MIMLh5 | (Taverniti et al., 2012) |
| TOVE R | Rat studies demonstrated competitive displacement of mutans streptococci and caries inhibition. | (Tanzer et al., 1985b, 1985a) |
|  | Interference with colonization of epithelial cells *in vitro* | (Sliepen et al., 2008, 2009) |
|  | Beagle dog model demonstrated TOVE R when administered together with *S.sanguinis* ATCC 49297 and *S. mitis* BMS interfered with gingival inflammation and pathogen recolonization | (Teughels et al., 2007) |
| F286 | Human breast milk isolate may exert regulatory (anti-inflammatory) role in gut and modulate the gut microbiota | (Li et al., 2022) |
| T30 | *In vitro* inhibitor of *S. pneumoniae, M. catarrhalis, H. influenzae and S. pyogenes* | (Wescombe et al., 2012) |

References

Bellussi, L. M., Villa, M. P., Degiorgi, G., Passali, F. M., Evangelisti, M., Paganelli, I. I., et al. (2018). Preventive nasal bacteriotherapy for the treatment of upper respiratory tract infections and sleep disordered breathing in children. *Int J Pediatr Otorhinolaryngol* 110, 43–47. doi: 10.1016/J.IJPORL.2018.04.024.

Birri, D. J., Brede, D. A., and Nes, I. F. (2012). Salivaricin D, a novel intrinsically trypsin-resistant lantibiotic from *Streptococcus salivarius* 5M6c isolated from a healthy infant. *Appl Environ Microbiol* 78, 402–410. doi: 10.1128/AEM.06588-11.

Geng, M., Deng, P., Mire, T., Austin, F., and Smith, L. (2019). Draft genome sequence of the lantibiotic-producing strain *Streptococcus salivarius* HS0302. *Microbiol Resour Announc* 8. doi: 10.1128/MRA.01410-18.

Gong, S.-G., Chan, Y., and Lévesque, C. M. (2019). Complete genome sequence of megaplasmid-bearing *Streptococcus salivarius* strain LAB813, isolated from the dental plaque of a caries-free child. *Microbiol Resour Announc* 8. doi: 10.1128/MRA.01092-19.

Guglielmetti, S., Taverniti, V., Minuzzo, M., Arioli, S., Stuknyte, M., Karp, M., et al. (2010). Oral bacteria as potential probiotics for the pharyngeal mucosa. *Appl Environ Microbiol* 76, 3948–3958. doi: 10.1128/AEM.00109-10.

Jörissen, J., van den Broek, M. F. L., de Boeck, I., van Beeck, W., Wittouck, S., Boudewyns, A., et al. (2021). Case-control microbiome study of chronic otitis media with effusion in children points at *Streptococcus salivarius* as a pathobiont-inhibiting species. *mSystems* 6. doi: 10.1128/MSYSTEMS.00056-21.

Kim, H., Fugaban, J. I. I., Holzapfel, W. H., and Todorov, S. D. (2022). Selection of beneficial bacterial strains with potential as oral probiotic candidates. *Probiotics Antimicrob Proteins* 14. doi: 10.1007/S12602-021-09896-Z.

Lawrence, G. W., Garcia-Gutierrez, E., Walsh, C. J., O’Connor, P. M., Begley, M., Cotter, P. D., et al. (2022a). Nisin G is a novel nisin variant produced by a gut-derived *Streptococcus salivarius*. *bioRxiv*, 2022.02.15.480493. doi: 10.1101/2022.02.15.480493.

Lawrence, G. W., McCarthy, N., Walsh, C. J., Kunyoshi, T. M., Lawton, E. M., O’Connor, P. M., et al. (2022b). Effect of a bacteriocin-producing *Streptococcus salivarius* on the pathogen *Fusobacterium nucleatum* in a model of the human distal colon. *Gut Microbes* 14. doi: 10.1080/19490976.2022.2100203.

Li, S., Li, N., Wang, C., Zhao, Y., Cao, J., Li, X., et al. (2022). Gut microbiota and immune modulatory properties of human breast milk *Streptococcus salivarius* and *S. parasanguinis* strains. *Front Nutr* 9, 798403. doi: 10.3389/fnut.2022.798403.

Manti, S., Parisi, G. F., Papale, M., Licari, A., Salpietro, C., Miraglia del Giudice, M., et al. (2020). Bacteriotherapy with *Streptococcus salivarius* 24SMB and *Streptococcus oralis* 89a nasal spray for treatment of upper respiratory tract infections in children: a pilot study on short-term efficacy. *Italian Journal of Pediatrics 2020 46:1* 46, 1–7. doi: 10.1186/S13052-020-0798-4.

Mantia, I. L. A., Varricchio, A., Girolamo, S. D. I., Minni, A., Passali, G. C., and Ciprandi, G. (2019). The role of bacteriotherapy in the prevention of adenoidectomy. *Eur Rev Med Pharmacol Sci* 23, 44–47. doi: 10.26355/EURREV_201903_17348.

Santagati, M., Scillato, M., Patanè, F., Aiello, C., and Stefani, S. (2012). Bacteriocin-producing oral streptococci and inhibition of respiratory pathogens. *FEMS Immunol Med Microbiol* 65, 23–31. doi: 10.1111/J.1574-695X.2012.00928.X.

Srikham, K., Daengprok, W., Niamsup, P., and Thirabunyanon, M. (2021). Characterization of *Streptococcus salivarius* as new probiotics derived from human breast milk and their potential on proliferative inhibition of liver and breast cancer cells and antioxidant activity. *Front Microbiol* 12. doi: 10.3389/FMICB.2021.797445.

Tarantino, V., Savaia, V., D’Agostino, R., Silvestri, M., and Ciprandi, G. (2018). Bacteriotherapy for preventing recurrent upper respiratory infections in children: a real-world experience. *Otolaryngol Pol* 72, 30–35. doi: 10.5604/01.3001.0012.0482.

Wescombe, P. A., Hale, J. D., Heng, N. C., and Tagg, J. R. (2012). Developing oral probiotics from *Streptococcus salivarius*. *Future Microbiol* 7, 1355–1371. doi: 10.2217/fmb.12.113.
