# Supplementary material for: Beneficial modulation of human health in the oral cavity and beyond using bacteriocin-like inhibitory substance-producing streptococcal probiotics
Source: Front Microbiol. 2023 Mar 28;14:1161155. doi: 10.3389/fmicb.2023.1161155 (PMC10086258; doi:10.3389/fmicb.2023.1161155)
Supplement: Supplementary file 4 [file Table_4.DOCX]

Supplementary table D

| **Application** | **Observation** | **References** |
| --- | --- | --- |
| *Streptococcus oligofermentans* AS 1.3089 | First description of novel species | (Tong et al., 2003) |
|  | Genome sequence | (Tong et al., 2013) |
|  | Factors influencing the competition between *Streptococcus oligofermentans* and *Streptococcus mutans* in dual-species biofilms. | (Bao et al., 2015, 2017) |
| *Streptococcus dentisani*  CECT 7746 | Defined as a novel member of the mitis group of streptococci | (Camelo-Castillo et al., 2014) |
|  | *Streptococcus dentisani* as an oral cavity niche specific oral probiotic | (López-López et al., 2017) |
|  | Increased the secretion of IL-10 and decreased the level of IFN-g induced  by *F. nucleatum* in HGF-1 (*in vitro* experiment) | (Esteban-Fernández et al., 2019) |
|  | Pilot oral colonization and pH buffering study | (Ferrer et al., 2020b) |
|  | Topic application improved various clinical and microbiological oral health parameters | (Ferrer et al., 2020a) |
|  | *Streptococcus dentisani* found at higher levels in caries-free individuals. | (López-Santacruz et al., 2021) |
| *S. oralis* 89a | Isolated from an individual seemingly resistant to streptococcal tonsillitis | (Grahn and Holm, 1983) |
|  | Member of a pool of four alpha-streptococci successfully used as supplementary treatment of recurrent streptococcal tonsillitis | (Roos et al., 1993) |
|  | Recurrences of acute otitis media and secretory otitis media reduced following treatment with consortium of 5 alpha streptococcus strains, including *S. oralis* 89a | (Roos et al., 2001) |
|  | Spray treatment showed some efficacy against secretory otitis media. | (Skovbjerg et al., 2009) |
|  | Draft genome sequence | (Sidjabat et al., 2016) |
| *S. sanguinis* BCC23 | Attenuated *S. mutans* oral and dental colonization and decreased severity of smooth surface caries. | (Culp et al., 2021) |
|  | Markedly reduced sulcal caries, persistently colonized mucosal and dental biofilms, and significantly lowered *S. mutans* counts in a mouse model | (Culp et al., 2022) |
| *S. parasanguinis* F298 | Human breast milk isolate showing potential for gut anti-inflammatory and microbiota  modulatory activity | (Li et al., 2022) |
| Streptococcus sp.A12 | A highly arginolytic Streptococcus that potently antagonizes *Streptococcus mutans* | (Huang et al., 2016) |
|  | Novel probiotic mechanisms explored using functional genomics*.* | (Lee et al., 2019) |
|  | A LanFEG-type ABC transporter enhances the ability of A12 to compete against *S. mutans*. | (Lee et al., 2021) |
| Probiora3 - containing  *Strep. oralis* strain KJ3sm,  *Strep. uberis* strain KJ2sm, and  *Strep. rattus*, strain JH145 | Preliminary assessment of the safety and effectiveness in humans of ProBiora3 | (Zahradnik et al., 2009) |
|  | No differences were detected in pocket probing depth, bleeding on probing, relative attachment levels and plaque and gingival indices associated with the adjunctive use for 12 weeks of a placebo or Probiora3 probiotic tablets | (Laleman et al., 2015) |
|  | Early childhood caries development may potentially be reduced via persistent application in a chewing tablet format | (Hedayati-Hajikand et al., 2015) |
| *Streptococcus mitis* 42885 | Producer of the bacteriocin viridin B | (Dajani et al., 1976) |
|  | Bactericidal to *Neisseria sicca* but bacteriostatic to a coagulase-negative staphylococcus | (Law and Dajani, 1978) |
| Streptococcus strain C17T | Speculated to have potential as an efficacious oropharyngeal probiotic | (Zhang et al., 2022) |
|  | Proposed novel species *Streptococcus symci,* similar to *Streptococcus pseudopneumoniae* | (Qi et al., 2021) |

References

Bao, X., de Soet, J. J., Tong, H., Gao, X., He, L., van Loveren, C., et al. (2015). *Streptococcus oligofermentans* inhibits *Streptococcus mutans* in biofilms at both neutral pH and cariogenic conditions. *PLoS One* 10, e0130962. doi: 10.1371/JOURNAL.PONE.0130962.

Bao, X., Yang, J., de Soet, J. J., Liu, H., Gao, X., van Loveren, C., et al. (2017). Factors influencing the competition between *Streptococcus oligofermentans* and *Streptococcus mutans* in dual-species biofilms. *Caries Res* 51, 507–514. doi: 10.1159/000479044.

Camelo-Castillo, A., Benítez-Páez, A., Belda-Ferre, P., Cabrera-Rubio, R., and Mira, A. (2014). *Streptococcus dentisani* sp. nov., a novel member of the mitis group. *Int J Syst Evol Microbiol* 64, 60–65. doi: 10.1099/IJS.0.054098-0.

Culp, D. J., Hull, W., Bremgartner, M. J., Atherly, T. A., Christian, K. N., Killeen, M., et al. (2021). *In Vivo* colonization with candidate oral probiotics attenuates *Streptococcus mutans* colonization and virulence. *Appl Environ Microbiol* 87. doi: 10.1128/AEM.02490-20.

Culp, D. J., Hull, W., Schultz, A. C., Bryant, A. S., Lizarraga, C. A., Dupuis, M. R., et al. (2022). Testing of candidate probiotics to prevent dental caries induced by *Streptococcus mutans* in a mouse model. *J Appl Microbiol* 132, 3853–3869. doi: 10.1111/JAM.15516.

Dajani, A. S., Tom, M. C., and Law, D. J. (1976). Viridins, bacteriocins of alpha-hemolytic streptococci: isolation, characterization, and partial purification. *Antimicrob Agents Chemother* 9, 81–88. doi: 10.1128/AAC.9.1.81.

Esteban-Fernández, A., Ferrer, M. D., Zorraquín-Peña, I., López-López, A., Moreno-Arribas, M. V., and Mira, A. (2019). In vitro beneficial effects of *Streptococcus dentisani* as potential oral probiotic for periodontal diseases. *J Periodontol* 90, 1346–1355. doi: 10.1002/JPER.18-0751.

Ferrer, M. D., López-López, A., Nicolescu, T., Perez-Vilaplana, S., Boix-Amorós, A., Dzidic, M., et al. (2020a). Topic application of the probiotic *Streptococcus dentisani* improves clinical and microbiological parameters associated with oral health. *Front Cell Infect Microbiol* 10, 465. doi: 10.3389/FCIMB.2020.00465/BIBTEX.

Ferrer, M. D., López-López, A., Nicolescu, T., Salavert, A., Méndez, I., Cuñé, J., et al. (2020b). A pilot study to assess oral colonization and pH buffering by the probiotic *Streptococcus dentisani* under different dosing regimes. *Odontology* 108, 180–187. doi: 10.1007/S10266-019-00458-Y.

Grahn, E., and Holm, S. E. (1983). Bacterial interference in the throat flora during a streptococcal tonsillitis outbreak in an apartment house area. *Zentralblatt für Bakteriologie, Mikrobiologie und Hygiene. 1. Abt. Originale. A, Medizinische Mikrobiologie, Infektionskrankheiten und Parasitologie* 256, 72–79. doi: 10.1016/S0174-3031(83)80054-7.

Hedayati-Hajikand, T., Lundberg, U., Eldh, C., and Twetman, S. (2015). Effect of probiotic chewing tablets on early childhood caries--a randomized controlled trial. *BMC Oral Health* 15. doi: 10.1186/S12903-015-0096-5.

Huang, X., Palmer, S. R., Ahn, S. J., Richards, V. P., Williams, M. L., Nascimento, M. M., et al. (2016). A highly arginolytic *Streptococcus* species that potently antagonizes *Streptococcus mutans*. *Appl Environ Microbiol* 82, 2187–2201. doi: 10.1128/AEM.03887-15.

Laleman, I., Yilmaz, E., Ozcelik, O., Haytac, C., Pauwels, M., Herrero, E. R., et al. (2015). The effect of a streptococci containing probiotic in periodontal therapy: a randomized controlled trial. *J Clin Periodontol* 42, 1032–1041. doi: 10.1111/JCPE.12464.

Law, D. J., and Dajani, A. S. (1978). Interactions between *Neisseria sicca* and viridin B, a bacteriocin produced by *Streptococcus mitis*. *Antimicrob Agents Chemother* 13, 473–478. doi: 10.1128/AAC.13.3.473.

Lee, K., Kaspar, J. R., Rojas-Carreño, G., Walker, A. R., and Burne, R. A. (2021). A single system detects and protects the beneficial oral bacterium *Streptococcus* sp. A12 from a spectrum of antimicrobial peptides. *Mol Microbiol* 116, 211–230. doi: 10.1111/MMI.14703.

Lee, K., Walker, A. R., Chakraborty, B., Kaspar, J. R., Nascimento, M. M., and Burne, R. A. (2019). Novel probiotic mechanisms of the oral bacterium *Streptococcus* sp. A12 as explored with functional genomics. *Appl Environ Microbiol* 85. doi: 10.1128/AEM.01335-19.

Li, S., Li, N., Wang, C., Zhao, Y., Cao, J., Li, X., et al. (2022). Gut microbiota and immune modulatory properties of human breast milk *Streptococcus salivarius* and *S. parasanguinis s*trains. *Front Nutr* 9, 798403. doi: 10.3389/fnut.2022.798403.

López-López, A., Camelo-Castillo, A., Ferrer, M. D., Simon-Soro, áurea, and Mira, A. (2017). Health-associated niche inhabitants as oral probiotics: The case of *Streptococcus dentisani.* *Front Microbiol* 8. doi: 10.3389/FMICB.2017.00379.

López-Santacruz, H. D., López-López, A., Revilla-Guarinos, A., Camelo-Castillo, A., Esparza-Villalpando, V., Mira, A., et al. (2021). *Streptococcus dentisani* is a common inhabitant of the oral microbiota worldwide and is found at higher levels in caries-free individuals. *Int Microbiol* 24, 619–629. doi: 10.1007/S10123-021-00222-9.

Qi, H., Liu, D., Zou, Y., Wang, N., Tian, H., and Xiao, C. (2021). Description and genomic characterization of *Streptococcus symci* sp. nov., isolated from a child’s oropharynx. *Antonie Van Leeuwenhoek* 114, 113–127. doi: 10.1007/S10482-020-01505-3.

Roos, K., Grahn Håkansson, E., and Holm, S. (2001). Effect of recolonisation with “interfering” alpha streptococci on recurrences of acute and secretory otitis media in children: randomised placebo controlled trial. *BMJ* 322, 210–212. doi: 10.1136/BMJ.322.7280.210.

Roos, K., Holm, S. E., Grahn, E., and Lind, L. (1993). Alpha-streptococci as supplementary treatment of recurrent streptococcal tonsillitis: a randomized placebo-controlled study. *Scand J Infect Dis* 25, 31–35. doi: 10.1080/00365549309169666.

Sidjabat, H. E., Håkansson, E. G., and Cervin, A. (2016). Draft genome sequence of the oral commensal *Streptococcus oralis* 89a with interference activity against respiratory pathogens. *Genome Announc* 4. doi: 10.1128/GENOMEA.01546-15.

Skovbjerg, S., Roos, K., Holm, S. E., Grahn Håkansson, E., Nowrouzian, F., Ivarsson, M., et al. (2009). Spray bacteriotherapy decreases middle ear fluid in children with secretory otitis media. *Arch Dis Child* 94, 92–98. doi: 10.1136/ADC.2008.137414.

Tong, H., Gao, X., and Dong, X. (2003). *Streptococcus oligofermentans* sp. nov., a novel oral isolate from caries-free humans. *Int J Syst Evol Microbiol* 53, 1101–1104. doi: 10.1099/IJS.0.02493-0.

Tong, H., Shang, N., Liu, L., Wang, X., Cai, J., and Dong, X. (2013). Complete genome sequence of an oral commensal, *Streptococcus oligofermentans* strain AS 1.3089. *Genome Announc* 1, 353–366. doi: 10.1128/GENOMEA.00353-13.

Zahradnik, R. T., Magnusson, I., Walker, C., McDonell, E., Hillman, C. H., and Hillman, J. D. (2009). Preliminary assessment of safety and effectiveness in humans of ProBiora3 ^TM^, a probiotic mouthwash. *J Appl Microbiol* 107, 682–690. doi: 10.1111/J.1365-2672.2009.04243.X.

Zhang, W. X., Xiao, C. L., Li, S. Y., Bai, X. C., Qi, H., Tian, H., et al. (2022). Streptococcus strain C17T as a potential probiotic candidate to modulate oral health. *Lett Appl Microbiol* 74, 901–908. doi: 10.1111/LAM.13680.
